# Metal Concentrations in Blood and Cerebrospinal Fluid of Patients With Arthroplasty Implants

**DOI:** 10.1001/jamanetworkopen.2025.2281

**Published:** 2025-03-28

**Authors:** Anastasia Rakow, Alexander Kowski, Sascha Treskatsch, Volker von Baehr, Claude L. Weynandt, Sascha Tafelski, Edda Klotz, Georg N. Duda, Carsten Perka, Katrin Huesker, Janosch Schoon

**Affiliations:** 1 Center for Musculoskeletal Surgery, Charité–Universitätsmedizin Berlin, corporate member of Freie Universität Berlin, Humboldt-Universität zu Berlin, and Berlin Institute of Health, Berlin, Germany; 2 Center for Orthopaedics, Trauma Surgery and Rehabilitation Medicine, University Medicine Greifswald, Greifswald, Germany; 3 Department of Neurology, Charité–Universitätsmedizin Berlin, corporate member of Freie Universität Berlin, Humboldt-Universität zu Berlin, and Berlin Institute of Health, Berlin, Germany; 4 Department of Anesthesiology and Intensive Care Medicine, Campus Benjamin Franklin, Charité–Universitätsmedizin Berlin, corporate member of Freie Universität and Humboldt Universität zu Berlin, Berlin, Germany; 5 Immunology Department, Institute for Medical Diagnostics (IMD), Berlin, Germany; 6 Department of Anaesthesiology and Intensive Care, Campus Charité Mitte and Campus Virchow-Klinikum, Charité–Universitätsmedizin Berlin, corporate member of Freie Universität Berlin, Humboldt-Universität zu Berlin, and Berlin Institute of Health, Berlin, Germany; 7 Julius Wolff Institute, Berlin Institute of Health at Charité—Universitätsmedizin Berlin, Berlin, Germany

## Abstract

**Importance:**

Arthroprosthetic metal exposure has been linked to neurological dysfunction, but evidence of central nervous system exposure is largely missing.

**Objective:**

To compare levels of all arthroplasty-relevant metals in cerebrospinal fluid (CSF), whole blood, and serum of patients with and without large joint replacement.

**Design, Setting, and Participants:**

The NeuroWear pilot study was a single-site hospital-based cross-sectional study conducted between April 2018 and November 2019. Laboratory personnel and investigators were blinded to group allocation. Patients presenting for elective surgery under spinal anesthesia and patients scheduled for lumbar puncture at a university medical center were eligible for inclusion. Patients aged 18 years and older with at least 1 large joint replacement in situ (103 case participants) and age- and sex-matched arthroplasty-naive patients (108 control participants) were recruited. One case and 6 control participants were excluded. Each case participant was matched with 1 control participant to achieve equal self-reported sex and a minimal age difference. The data analyses were performed between May 2023 and February 2024.

**Exposure:**

Presence of large joint replacement.

**Main Outcomes and Measures:**

CSF, whole blood, and serum levels of aluminum, cobalt, chromium, molybdenum, nickel, niobium, tantalum, titanium, vanadium, and zirconium were quantified.

**Results:**

A total of 204 patients (118 [58%] women and 86 [42%] men; median [range] age 69.4 [21.3-93.1] years) were included for metal analyses. In CSF, median (range) cobalt levels were significantly higher in the implant group (0.03 [0.01-0.64] μg/L) compared with the control group (0.02 [0.01-0.19] μg/L). Cobalt levels in CSF were significantly correlated with cobalt levels in serum (*r* = 0.72; 95% CI, 0.53-0.85) and whole blood (*r* = 0.82; 95% CI, 0.62-0.92). Significantly higher whole blood median (range) levels of cobalt (implant: 0.27 [0.07-24.10] μg/L; control: 0.16 [0.08-0.99] μg/L), chromium (implant: 0.47 [0.24-4.76] μg/L; control: 0.42 [0.21-1.52] μg/L), titanium (implant: 8.05 [1.14-37.20] μg/L; control: 7.15 [1.80-20.70] μg/L), niobium (implant: 0.02 [0.01-1.14] μg/L; control: 0.01 [0.01-0.11] μg/L), and zirconium (implant: 0.05 [0.01-39.90] μg/L; control: 0.03 [0.01-1.95] μg/L) were detected. Patients with an implant were found to have significantly higher median (range) CSF metal levels for titanium (implant: 0.75 [0.12-1.40] μg/L; control: 0.57 [0.13-1.10] μg/L), niobium (implant: 0.02 [0.01-0.16] μg/L; control: 0.01 [0.01-0.03] μg/L), and zirconium (implant: 0.05 [0.01-0.44] μg/L; control: 0.04 [0.01-0.28] μg/L) if those metal levels were elevated in serum. Patients with cobalt-chromium-molybdenum implant components exhibited significantly higher median (range) chromium levels in CSF than control participants (implant: 0.31 [0.02-2.05] μg/L; control: 0.23 [0.02-1.10] μg/L).

**Conclusions and Relevance:**

In this cross-sectional study, chronic exposure to arthroplasty implants was associated with corresponding metal accumulation in blood and CSF. Arthroprosthetic cobalt, chromium, titanium, niobium, and zirconium seemed to cross neural barriers and accumulate in CSF. Correlation analyses suggested cobalt-specific transport mechanisms across neural barriers. In view of the neurotoxic effects of cobalt, subsequent studies are needed to determine whether CSF metal concentrations correlate with objective measures of neurotoxic effects and whether this may be of relevance, particularly in patients with new-onset or rapid deterioration of neurological conditions following arthroplasty.

## Key Points

**Question:**

Do patients with large joint replacements have elevated levels of arthroplasty-relevant metals in blood and cerebrospinal fluid (CSF)?

**Findings:**

This cross-sectional study with 204 patients found blood levels of cobalt, chromium, titanium, niobium, and zirconium to be significantly higher in patients with large joint replacements compared with arthroplasty-naive control participants. Cobalt levels in CSF and blood were significantly correlated; although not correlated, chromium, titanium, niobium, and zirconium accumulated in CSF of participants with arthroplasty implants.

**Meaning:**

These findings suggest that arthroplasty implant–related systemic and central nervous system exposure to various metals should be considered a contributory factor in postarthroplasty adverse events, including neurotoxic effects.

## Teaser

This cross-sectional study compares the levels of aluminum, cobalt, chromium, molybdenum, nickel, niobium, tantalum, titanium, vanadium, and zirconium in the cerebrospinal fluid and blood of individuals in Germany with and without large joint replacements.

## Introduction

Arthroplasty is a cornerstone in contemporary surgical history, continuing to improve the quality of life of millions of patients around the world every year.^1,2,3,4^ The vast success of total hip arthroplasty has contributed to the broadening of the spectra of indications for joint replacements and of patients undergoing such surgeries.^1,2,5^ Longevity and biocompatibility of the implants used to replace the troublesome joints are of utmost importance to every patient in need of arthroplasty, from women of child-bearing age with immobilizing secondary osteoarthritis (OA) of the knee to athletically ambitious best-agers with end-stage primary OA of the shoulder and multimorbid octogenarians with a femoral neck fracture.^5,6^

However, in the human body, all arthroplasty implants are subject to tribological and biological processes, resulting in wear and corrosion of the implant components.^7,8^ Respective degradation products have repeatedly been shown to negatively impact bone and soft tissues surrounding the prosthesis,^9,10,11^ contributing to implant loosening, joint instability, and possibly to other modes of implant failure, including periprosthetic infection.

In recent years, increasing concern has been raised regarding potential systemic toxic effects of metals released from arthroplasty implants.^12^ Several case reports and series linked systemic arthroprosthetic cobalt exposure to significant cardiac, thyroid, and/or neurological dysfunction,^13,14,15,16,17,18^ and rare experimental studies and analyses of arthroplasty registries focused on the potential immunotoxic effects, cancerogenicity, and teratogenicity of cobalt and chromium species.^19,20,21,22^ Of note, those data have been collected mainly from patients with hip replacements with so-called metal-on-metal (MoM) bearings and have mostly been limited to analyses of cobalt and chromium serum levels. Studies on structural changes in the brain as a function of bloodstream exposure to cobalt and chromium released from arthroplasty implants show contradictory results.^23,24^ The prospect of multidecade survival of a multitude of modern arthroplasty implants, many of which are made of up to 10 metals; a lack of valid metal thresholds for human (organ) toxic effects; and the uncertainty regarding possible systemic effects of respective long-term exposure scenarios pose special challenges.^12,25^

Given hints of evidence of increasing prevalence of neurodegenerative diseases and psychiatric disorders in patients with arthroplasty implants,^22,26,27^ and the proven neurotoxic effects of metals widely used in arthroplasty, such as titanium, cobalt, and vanadium,^28,29,30^ investigating the exposure of the human central nervous system (CNS) to arthroprosthetic metal degradation products appears especially relevant. Thus, the purpose of this study was to determine whether and to what extent chronic exposure to wear and corrosion products of arthroplasty implants is associated with metal accumulation in cerebrospinal fluid (CSF).

## Methods

### Design

The NeuroWear pilot study was a single-center hospital-based cross-sectional study prospectively including patients at the Charité–Universitätsmedizin Berlin, Germany, between April 2018 and November 2019. The study was designed and conducted in compliance with the principles of the Declaration of Helsinki and in accordance with the Good Clinical Practice guidelines of the International Conference for Harmonisation. The protocol, consent form, and other relevant documents were reviewed and approved by the institutional data protection officers and the independent ethics committee of the Charité–Universitätsmedizin Berlin. The study was prospectively registered with the German Clinical Trials Register (DRKS) and the World Health Organization’s International Clinical Trials Registry Platform (Identifiers: DRKS00014555 and DRKS00014556). All participants provided written informed consent before study entry. The NeuroWear pilot study was reported following the Strengthening the Reporting of Observational Studies in Epidemiology (STROBE) reporting guideline for cross-sectional studies.

### Participants and Matching

Patients with at least 1 large artificial joint (ie, an arthroplasty implant of the hip, knee, ankle, shoulder, or elbow joint) in situ were recruited for the implant group. Age- and sex-matched arthroplasty-naive patients served as control group. Eligible participants were adults (aged ≥18 years) who presented to hospital-based surgical departments to undergo elective surgery under spinal anesthesia (SPA) (NeuroWear I) or adult patients scheduled for lumbar puncture (LP) in the course of routine diagnostics or therapy at the department of neurology (NeuroWear II). Potential participants were excluded if contraindications of SPA or LP were prevalent. A complete list of inclusion and exclusion criteria is detailed in the supplement (eAppendix 1 in Supplement 1). Each case participant was matched with 1 control participant for statistical comparison. Data for 1 implant group patient were not analyzed due to excessive storage time at 4 °C, and data for 6 patients in the control group were not analyzed due to insufficient sample volume, drained CSF tube, missing CSF or serum sample, missing corresponding S100-B level, and excessive storage time at 4 °C. Retrospective matching resulted in an equal self-reported sex distribution (female, male, and unspecified). Criteria for matching were to achieve the minimal age difference between the 2 groups (4.7-year mean age difference; mean [SD] age of implant group, 71.3 [10.5] years; control group, 66.6 [12.3] years). The respective matching of the study participants, along with their age and self-reported sex, as well as the study participants whose samples were not analyzed, are listed in eTables 1 and 2 in Supplement 1.

### Procedures

Standard cannulas for blood and CSF collection had previously been tested for any release of metals relevant to this study to exclude contaminations and were used with trace element–free vacutainer tubes. Multimetal quantifications in CSF, whole blood (WB), and serum of all participants were performed by inductively coupled plasma mass spectrometry (ICP-MS). Results represent means of 3 measurements each. Serum levels of S-100B protein were analyzed by an automated chemiluminescent immunoassay (CLIA) (Liaison s100, Diasorin) according to the manufacturer’s instructions. All laboratory investigators and staff were blinded to group allocation. Details on procedures and data collection, including the case report forms used, are provided in the supplement (eAppendices 2-4 in Supplement 1).

### Outcomes

Primary end points were the concentrations of aluminum, cobalt, chromium, molybdenum, nickel, niobium, tantalum, titanium, vanadium, and zirconium in CSF. Secondary end points include the CSF-to-WB ratio and the CSF-to-serum ratio of the aforementioned metals, and the integrity of the blood-CNS barrier (S-100B level in serum) in patients exposed to arthroplasty metals and control patients.

### Statistical Analysis

Due to the novel and exploratory character of this pilot study, sample size was not predetermined. The recruitment target was 100 participants, 50 per group, at minimum. The curation and subsequent analysis of the data were conducted between May 2023 and February 2024. Exploratory and descriptive statistical analyses and data plotting were conducted using Prism version 8.4.3 (GraphPad). The Shapiro-Wilk test was conducted to assess normal distribution. Two-tailed Wilcoxon matched-pairs signed-rank test was used to compare metal levels, age, and body mass index. Two-tailed Mann-Whitney test was used to compare tobacco smoking history (pack-years). Two-sided Fisher exact test was used to compare concomitant diseases between the groups. To examine the association between nonnormally distributed variables, we used 2-tailed Spearman rank correlation. The significance level (α) was set at .05. If multiple comparisons in the course of correlation analyses were applied, the *P* value was adjusted according to Bonferroni.

## Results

A total of 204 patients (118 [58%] women and 86 [42%] men; median [range] age 69.4 [21.3-93.1] years) were included in this study, with 102 assigned to each the implant group and the control group. Arthroplasty data for the implant group are summarized in the Table. Baseline and medical characteristics at enrollment were similar between groups (Table). Of note, patients in the implant group were significantly older than control participants (median [range] age, 71.7 [38.6-88.9] years vs 67.2 [21.3-93.1] years; *P* < .001). Spearman correlation analyses between metal levels and age of the control group revealed a significant positive correlation between age and molybdenum levels in WB and a significant negative correlation between age and vanadium levels in serum (eTable 3 in Supplement 1).

**Table.** Patient Demographic Characteristics and Implant Data

Patient characteristics	Patients, No. (%)			*P* value
	Study population (N = 204)	Implant group (n = 102)	Control group (n = 102)	
Age, median (range), y	69.4 (21.3-93.1)	71.7 (38.6-88.9)	67.2 (21.3-93.1)	<.001
Self-reported sex				
Female	118 (57.8)	59 (57.8)	59 (57.8)	NA
Male	86 (42.2)	43 (42.2)	43 (42.2)	
Unspecified	0	0	0	
BMI, median (range)	27.7 (15.4-57.4)	27.8 (20.4-57.4)	27.6 (15.4-43.3)	.92
ASA classification				
I	9 (4.4)	3 (2.9)	6 (5.9)	.50
II	98 (48.0)	50 (49.0)	48 (47.1)	.89
III	47 (23.0)	26 (25.5)	21 (20.6)	.51
IV	1 (0.5)	0	1 (1.0)	>.99
NA	49 (24.0)	23 (22.5)	26 (25.5)	.74
Concomitant diseases				
Neurological disorders				
Any	67 (32.8)	33 (32.4)	34 (33.3)	>.99
History of stroke(s)	11 (5.4)	3 (1.5)	8 (3.9)	
Normal pressure hydrocephalus	5 (2.5)	1 (0.5)	4 (2.0)	
Restless legs syndrome	5 (2.5)	2 (1.0)	3 (1.5)	
Motor neurone disease	11 (5.4)	3 (1.5)	8 (3.9)	
Amyotrophic lateral sclerosis, confirmed	4 (2.0)	1 (0.5)	3 (1.5)	
Amyotrophic lateral sclerosis, suspected	5 (2.5)	1 (0.5)	4 (2.0)	
Hereditary spastic paraplegia	1 (0.5)	1 (0.5)	0	
Unspecified motor neurone disease	1 (0.5)	0	1 (0.5)	
Polyneuropathy	15 (7.4)	9 (4.4)	6 (2.9)	
Epilepsy	6 (2.9)	3 (1.5)	3 (1.5)	
Migraine	4 (2.0)	2 (1.0)	2 (1.0)	
Parkinson disease	3 (1.5)	2 (1.0)	1 (0.5)	
Other	36 (17.6)	20 (9.8)	16 (7.8)	
Dementia (any type)	13 (6.4)	7 (6.9)	6 (5.9)	>.99
Psychiatric disorders				
Any	29 (14.2)	17 (16.7)	12 (11.8)	.42
Major depressive disorder	24 (11.8)	14 (13.7)	10 (9.8)	
Anxiety disorder	7 (3.4)	3 (2.9)	4 (3.9)	
Other	4 (2.0)	2 (2.0)	2 (2.0)	
Rheumatic diseases				
Any	22 (10.8)	12 (11.8)	10 (9.8)	.82
Rheumatoid arthritis	9 (4.4)	7 (6.9)	2 (2.0)	
Psoriasis arthritis	1 (0.5)	1 (1.0)	0	
Ankylosing spondylitis/Bechterew disease	1 (0.5)	1 (1.0)	0	
Gout	8 (3.9)	1 (1.0)	7 (6.9)	
CREST syndrome	1 (0.5)	1 (1.0)	0	
SLE	1 (0.5)	1 (1.0)	0	
Diabetes				
Any	37 (18.1)	28 (27.5)	9 (8.8)	.47
NIDD	16 (7.8)	12 (11.8)	4 (3.9)	
IDD	21 (10.3)	16 (15.7)	5 (4.9)	
Hyperlipidaemia	60 (29.4)	32 (31.4)	28 (27.5)	.65
Thyroid disease or dysfunction				
Any	49 (24.0)	21 (20.6)	28 (27.5)	.33
Hypothyroidism	46 (22.5)	21 (20.6)	25 (24.5)	
Hashimoto disease	2 (1.0)	0	2 (2.0)	
Nodular goiter	1 (0.5)	0	1 (1.0)	
Cardiovascular diseases				
Any	139 (68.1)	69 (67.6)	70 (68.6)	>.99
Arterial hypertension	131 (64.2)	66 (64.7)	65 (63.7)	
Coronary artery disease	29 (14.2)	14 (13.7)	15 (14.7)	
Heart valve disease(s)	14 (6.9)	9 (8.8)	5 (4.9)	
Chronic heart failure	21 (10.3)	10 (9.8)	11 (10.8)	
Cardiac rhythm disorder	24 (11.8)	13 (12.7)	11 (10.8)	
Cardiac conduction disorder	11 (5.4)	7 (6.9)	4 (3.9)	
Peripheral artery disease	3 (1.5)	2 (2.0)	1 (1.0)	
Carotid artery disease	7 (3.4)	0	7 (6.9)	
History of DVT or PE	15 (7.4)	11 (10.8)	4 (3.9)	
Other	15 (7.4)	7 (6.9)	8 (7.8)	
Pulmonary diseases				
Any	24 (11.8)	12 (11.8)	12 (11.8)	>.99
Asthma	11 (5.4)	8 (7.8)	3 (2.9)	
Chronic obstructive pulmonary disease	8 (3.9)	5 (4.9)	3 (2.9)	
Posttuberculosis lung disease	2 (1.0)	0	2 (2.0)	
History of lung cancer or lobectomy due to lung cancer	2 (1.0)	1 (1.0)	1 (1.0)	
Neuromuscular hypoventilation syndrome	3 (1.5)	0	3 (2.9)	
Mixed obstructive and restrictive ventilatory defect	2 (1.0)	2 (2.0)	0	
Kidney diseases	25 (12.3)	15 (14.7)	10 (9.8)	
Any				.39
Chronic kidney disease	15 (7.4)	8 (7.8)	7 (6.9)	
Hyperuricemia	7 (3.4)	4 (3.9)	3 (2.9)	
Nephrostomy following urothelial carcinoma	1 (0.5)	0	1 (1.0)	
Status post kidney transplantation following SLE	1 (0.5)	1 (1.0)	0	
Nephrolithiasis	1 (0.5)	1 (1.0)	0	
Congenital solitary kidney	1 (0.5)	1 (1.0)	0	
Renal cysts	1 (0.5)	1 (1.0)	0	
Liver or biliary diseases				
Any	11 (5.4)	7 (6.9)	4 (3.9)	.54
Nonalcoholic fat liver disease	2 (1.0)	0	2 (2.0)	
NASH or posthepatitis NASH	5 (2.5)	3 (2.9)	2 (2.0)	
Polycystic liver disease	1 (0.5)	1 (1.0)	0	
Status post cholecystectomy	4 (2.0)	4 (3.9)	0	
Chronic cholangitis	1 (0.5)	1 (1.0)	0	
Hepatitis A	1 (0.5)	1 (1.0)	0	
Status post liver transplantation due to HCC and cirrhosis	1 (0.5)	1 (1.0)	0	
Known active malignant neoplasm				
Any	7 (3.4)	1 (1.0)	6 (5.9)	.21
Lung	1 (0.5)	0	1 (1.0)	
Breast	1 (0.5)	0	1 (1.0)	
Prostate	2 (1.0)	1 (1.0)	1 (1.0)	
Bladder or urothelial	2 (1.0)	0	2 (2.0)	
Esophageal (squamous-cell carcinoma)	1 (0.5)	0	1 (1.0)	
History of previous malignant neoplasm				
Any	22 (10.8)	14 (13.7)	8 (7.8)	.26
Breast	3 (1.5)	3 (2.9)	0	
Prostate	5 (2.5)	3 (1.5)	2 (2.0)	
Bladder and/or urothelial	3 (1.5)	1 (1.0)	2 (2.0)	
Endometrial	2 (1.0)	1 (1.0)	1 (1.0)	
Melanoma	2 (1.0)	2 (2.0)	0	
Basal cell carcinoma	1 (0.5)	0	1 (1.0)	
Merkel cell carcinoma	1 (0.5)	0	1 (1.0)	
Renal cell carcinoma	1 (0.5)	0	1 (1.0)	
Gastric	1 (0.5)	1 (1.0)	0	
Colorectal	2 (1.0)	2 (2.0)	0	
HCC	1 (0.5)	1 (1.0)	0	
Pack-years, median (range)	0 (0-60)	0 (0-47)	0 (0-60)	.07
Smoking status, No. (%)				
Never smoked	150 (73.5)	80 (78.4)	70 (68.6)	.15
Current smoker	17 (8.3)	6 (7.8)	11 (10.8)	.31
Former smoker	37 (18.1)	16 (15.7)	21 (20.6)	.47
History of relevant allergies and nonarthroplasty metal exposure				
Self-reported history of any (including non-metal) allergic contact dermatitis	22 (10.8)	10 (9.8)	12 (11.8)	.82
Confirmed metal hypersensitivity and/or allergy				
Any	10 (4.9)	5 (4.9)	5 (4.9)	>.99
Cobalt	1 (0.5)	1 (1.0)	0	
Nickel	7 (3.4)	2 (2.0)	5 (4.9)	
Copper	1 (0.5)	1 (1.0)	0	
Mercury	1 (0.5)	1 (1.0)	0	
Zinc	1 (0.5)	1 (1.0)	0	
History of occupational metal exposure^a^	13 (6.4)	6 (5.9)	7 (6.9)	>.99
Piercings in situ at inclusion	29 (14.2)	14 (13.7)	15 (14.7)	>.99
Tattoos	6 (2.9)	3 (2.9)	3 (2.9)	>.99
Nonarthroplasty metal implants in situ				
Any	71 (34.8)	33 (32.4)	38 (37.3)	.56
Dentures	36 (17.6)	15 (14.7)	21 (20.6)	
Osteosynthesis and/or arthrodesis implants	21 (10.3)	14 (13.7)	7 (6.9)	
Spinal instrumentation	2 (1.0)	1 (1.0)	1 (1.0)	
Spinal cage	1 (0.5)	0	1 (1.0)	
History of kyphoplasty	1 (0.5)	1 (1.0)	0	
Metal suture material (musculoskeletal, intraabdominal)	3 (1.5)	1 (1.0)	2 (2.0)	
Metal-containing mesh graft (abdominal/inguinal)	1 (0.5)	1 (1.0)	0	
Cardiac or vascular implantable devices				
Any	16 (7.8)	5 (4.9)	11 (10.8)	
Mechanical valve replacement	2 (1.0)	1 (1.0)	1 (1.0)	
Pacemaker	3 (1.5)	2 (2.0)	1 (1.0)	
Implantable cardioverter defibrillator	3 (1.5)	3 (2.9)	0	
Cardiac stent	11 (5.4)	0	11 (10.8)	
Noncardiac vascular stent	1 (0.5)	1 (1.0)	0	
Bile duct stent	1 (0.5)	1 (1.0)	0	
Arthroplasty implants in situ at sampling, No.				
1	68 (33.3)	68 (66.7)	0	NA
2	29 (14.2)	29 (28.4)	0	NA
3	5 (2.5)	5 (4.9)	0	NA
Total arthroplasty implants in situ at sampling, No./total No. (%)				
Total	141/141 (100)	141/141 (100)	0	NA
Hip	69/141 (48.9)	69/141 (48.9)	0	NA
Knee	70/141 (49.6)	70/141 (49.6)	0	NA
Shoulder	2/141 (1.4)	2/141 (1.4)	0	NA
Time since first large joint arthroplasty implantation, median (range), y	NA	9.68 (0.02-40.90)	NA	NA
Years since first large joint arthroplasty implantation				
<1	11 (5.4)	11 (10.8)	0	NA
<3	13 (6.4)	13 (12.7)	0	NA
<6	13 (6.4)	13 (12.7)	0	NA
<10	15 (7.4)	15 (14.7)	0	NA
<15	24 (11.8)	24 (23.5)	0	NA
<20	16 (7.8)	16 (15.7)	0	NA
≥20	10 (4.9)	10 (9.8)	0	NA
Components in situ				
Cobalt-chromium-molybdenum	68 (33.3)	68 (66.7)	0	NA
Ceramic	35 (17.2)	35 (34.3)	0	NA
Bone cement	62 (30.4)	62 (60.8)	0	NA
Tantalum	4 (2.0)	4 (3.9)	0	NA
PMMA bone cement spacer history, No.				
1	5 (2.5)	5 (4.9)	0	NA
2	2 (1.0)	2 (2.0)	0	NA
3	2 (1.0)	2 (2.0)	0	NA
Lifetime tribological pairings, No.				
1	50 (24.5)	50 (49.0)	0	NA
2	26 (12.7)	26 (25.5)	0	NA
3	12 (5.9)	12 (11.8)	0	NA
4	6 (2.9)	6 (5.9)	0	NA
5	3 (1.5)	3 (2.9)	0	NA
6	3 (1.5)	3 (2.9)	0	NA
7	3 (1.5)	3 (2.9)	0	NA
8	1 (0.5)	1 (1.0)	0	NA
Previous arthroplasty implant revisions at sampling, No.				
1	24 (11.8)	24 (23.5)	0	NA

Abbreviations: ASA, American Society of Anesthesiologists; BMI, body mass index (calculated as weight in kilograms divided by height in meters squared); DVT, deep vein thrombosis; HCC, hepatocellular carcinoma; IDD, insulin-dependent diabetes; NA, not applicable; NASH, nonalcoholic steatohepatitis; NIDD, non–insulin-dependent diabetes; PE, pulmonary embolism; PMMA, polymethylmethacrylate; SLE, systemic lupus erythematosus.

^a^ Among the occupationally exposed participants in the implant group, there was 1 dentist, 1 agricultural engine machinist, 1 aircraft machinist, 1 steel mill worker, 1 building locksmith, and 1 sheet metal worker. Among those in the control group, there was 1 dentist, 1 bridge-builder, 1 copperworks worker, 1 heating engineer, and 3 sheet metal workers. All these occupationally exposed study participants presented with an occupational medicine record suggesting no critical work-space exposure to the metals relevant in arthroplasty.

**Figure 1.**
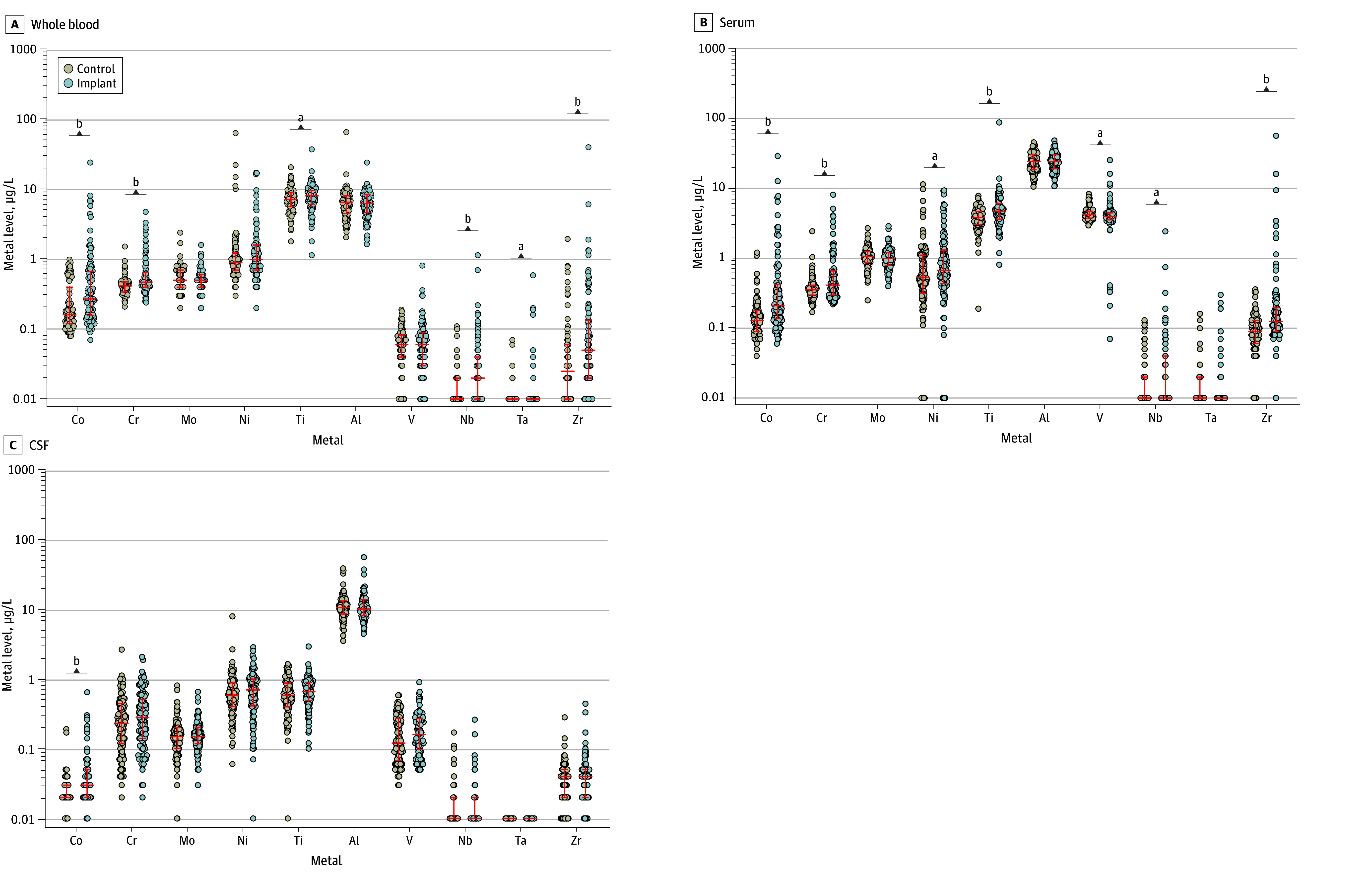
Multimetal Quantification in Whole Blood, Serum and Cerebrospinal Fluid (CSF) of 102 Patients With at Least 1 Arthroplasty Implant In Situ and 102 Patients Without Levels of quantified arthroplasty metals. Red middle lines indicate median, with whiskers indicating IQRs. Wilcoxon matched-pairs signed rank test was used to test statistical difference. Al indicates aluminum; Co, cobalt; Cr, chromium; Mo, molybdenum; Nb, niobium; Ni, nickel; Ta, tantalum; Ti, titanium; V, vanadium; and Zr, zirconium. ^a^*P* < .05. ^b^*P* < .001.

Multimetal quantification revealed that the implant group, compared with the control group, had significantly higher WB levels of cobalt (implant: 0.27 [0.07-24.10] μg/L; control: 0.16 [0.08-0.99] μg/L), chromium (implant: 0.47 [0.24-4.76] μg/L; control: 0.42 [0.21-1.52] μg/L), titanium (implant: 8.05 [1.14-37.20] μg/L; control: 7.15 [1.80-20.70] μg/L), niobium (implant: 0.02 [0.01-1.14] μg/L; control: 0.01 [0.01-0.11] μg/L), tantalum (implant: 0.01 [0.01-0.59] μg/L; control: 0.01 [0.01-0.07] μg/L) and zirconium (implant: 0.05 [0.01-39.90] μg/L; control: 0.03 [0.01-1.95] μg/L). Serum analyses showed that the implant group had significantly higher levels of cobalt, chromium, nickel, titanium, vanadium, niobium, and zirconium compared with control participants. In CSF, cobalt levels were significantly higher in patients of the implant group (0.03 [0.01-0.64] μg/L) than those in the control group (0.02 [0.01-0.19] μg/L) (Figure 1). The quantified metal levels in WB, serum, and CSF of all patients are depicted in eTables 4 to 6 in Supplement 1. Median and maximum metal levels of both groups and respective *P* values appear in eTable 7 in Supplement 1. Subdivision of the implant group and comparison with the matched control participants revealed that patients with at least 1 cobalt-chromium-molybdenum component had significantly higher levels of cobalt and chromium in WB, serum, and CSF (eg, chromium in CSF: implant, 0.31 [0.02-2.05] μg/L; control, 0.23 [0.02-1.10] μg/L) (eTable 7 in Supplement 1). Further analyses of this group revealed that cobalt levels in CSF were significantly higher in patients with implants in situ for both more than and less than 10 years (eFigure 1A in Supplement 1). Patients who reported pain in the index joint at the time of sampling exhibited significantly higher cobalt levels in the CSF. This significant difference was not observed in patients with a pain-free index joint (eFigure 1B in Supplement 1). Patients with cobalt-chromium-molybdenum–free arthroplasty implants had significantly higher titanium levels in serum and nickel levels in whole blood compared with control participants. However, no metals were found to be significantly higher in the CSF of those patients. Patients with arthroplasty implants without cobalt-chromium-molybdenum components did not exhibit significantly higher levels of cobalt or chromium in any of the biological matrices analyzed (eTable 7 in Supplement 1). Stratification of the implant group based on the individual index arthroplasty implants indicated that patients with knee arthroplasty implants were particularly exposed to cobalt in the bloodstream. However, both patients with hip arthroplasty implants and those with knee arthroplasty implants exhibited significantly higher cobalt levels in CSF compared with control participants (eFigure 2 in Supplement 1).

**Figure 2.**
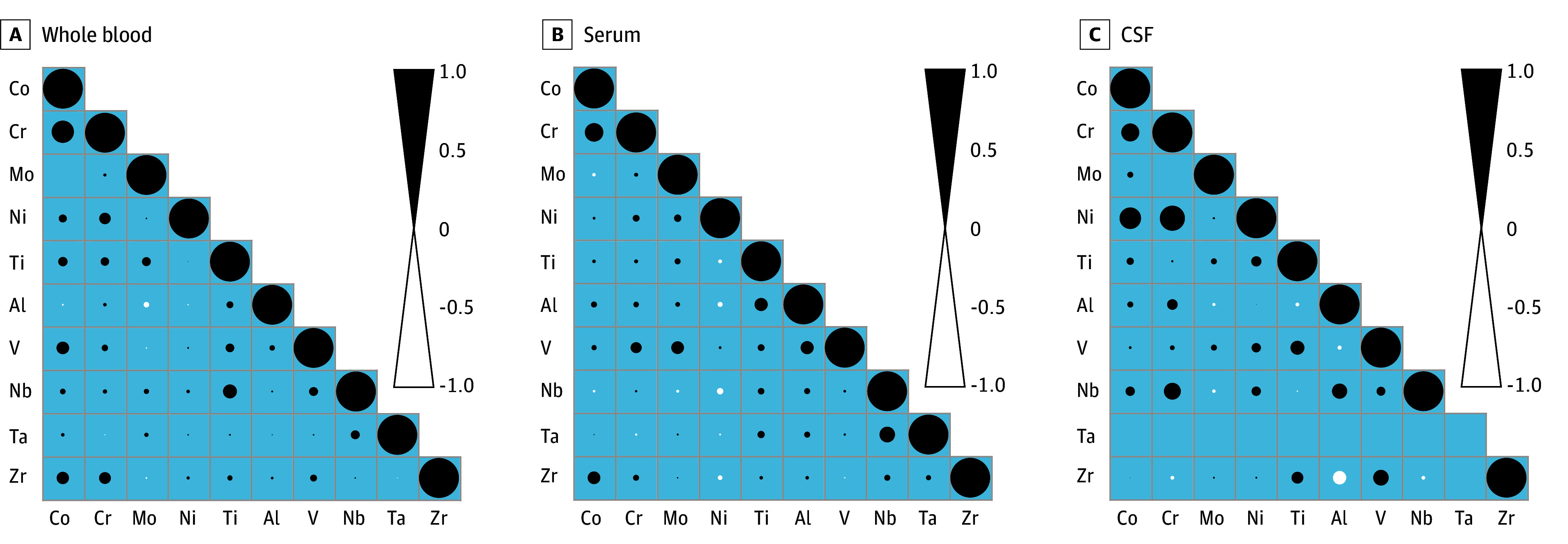
Correlation Matrix of Metal Levels Quantified in Whole Blood, Serum, and Cerebrospinal Fluid (CSF) of Patients With at Least 1 Arthroplasty Implant In Situ Black and white circles indicate the Spearman correlation coefficient between each 2 arthroplasty metals, ie, their levels within whole blood, serum, and CSF. Al indicates aluminum; Co, cobalt; Cr, chromium; Mo, molybdenum; Nb, niobium; Ni, nickel; Ta, tantalum; Ti, titanium; V, vanadium; and Zr, zirconium.

To assess correlations of metal levels and alloy constituents of the implant materials cobalt-chromium-molybdenum, titanium-aluminum-vanadium, and titanium-aluminum-niobium, correlation coefficients of these variables were determined in the respective biological matrix (Figure 2). The correlation matrix of metal levels in WB revealed significant correlations between cobalt and chromium in WB (*r* = 0.55; 95% CI, 0.40-0.68), serum (*r* = 0.46; 95% CI, 0.29-0.61), and CSF (*r* = 0.46; 95% CI, 0.28-0.60), while molybdenum levels did not correlate with cobalt and chromium levels. Titanium levels in WB were significantly correlated with niobium levels. Titanium and aluminum levels were significantly correlated in serum. Zirconium levels were significantly correlated with cobalt levels in WB and serum, but not in CSF. The respective *P* values of the intramatrix correlation analyzes are depicted in eTables 8 to 10 in Supplement 1.

**Figure 3.**
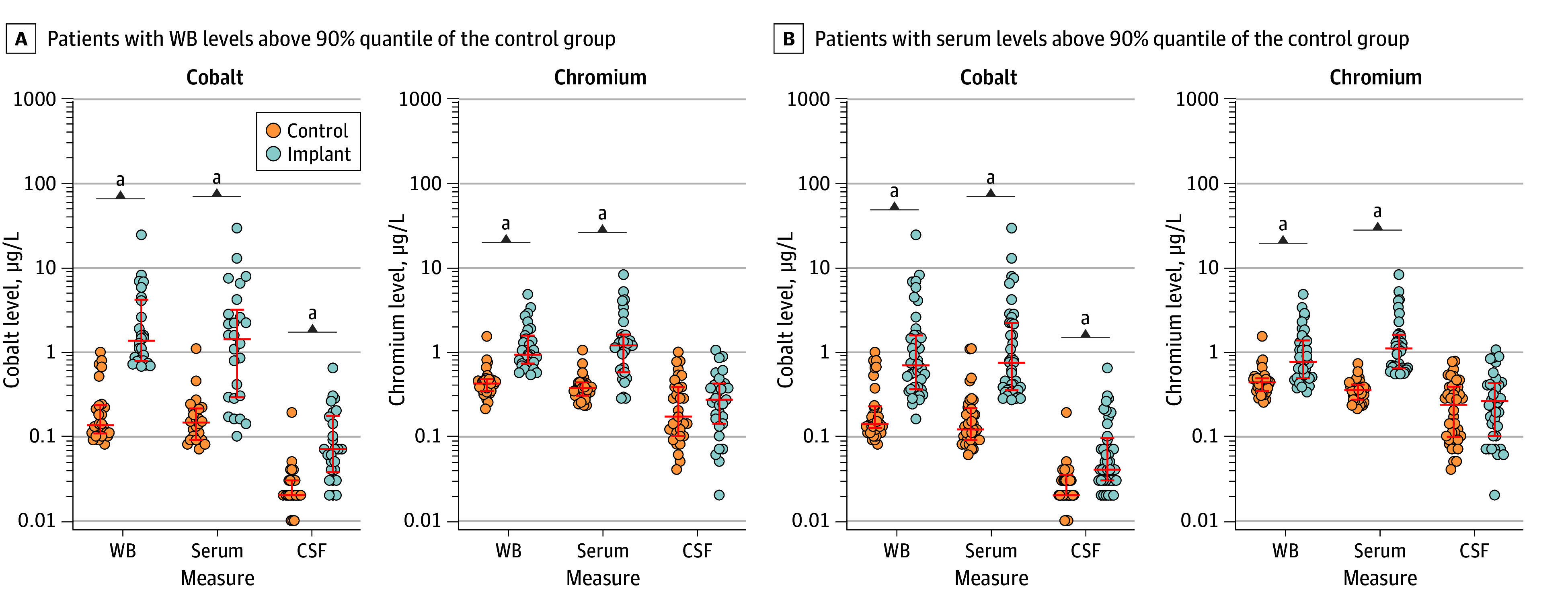
Metal Levels of Patients in the Implant Group With Elevated Metals in Whole Blood and/or Serum and the Corresponding Levels in Cerebrospinal Fluid Red middle lines indicate median, with whiskers indicating IQRs. A, A total of 26 patients and 31 patients in the implant group had whole blood cobalt and chromium levels, respectively, greater than the 90% quantile in the control group. B, A total of 41 patients and 38 patients in the implant group had serum cobalt and chromium levels, respectively, greater than the 90% quantile in the control group. Wilcoxon matched-pairs signed rank test was used to test statistical difference. ^a^*P* < .001.

The levels of cobalt, chromium, titanium, niobium, and zirconium were found to be significantly higher in WB and serum of patients in the implant group compared with controls. Comparative analyses of metal levels in the different biological matrices showed significantly higher median (range) levels of cobalt in CSF of patients in the implant group with elevated cobalt levels in WB compared with individuals in the control group (implant: 0.07 [0.02-0.64] μg/L; control: 0.02 [0.01-0.19] μg/L; *P* < .001) or serum (implant: 0.04 [0.02-0.64] μg/L; control: 0.02 [0.01-0.19] μg/L; *P* < .001) (Figure 3). In patients with elevated chromium levels in WB and/or serum, significantly different chromium levels in CSF were not detected. Patients with elevated levels of titanium, niobium, or zirconium in WB did not exhibit elevated levels of the respective metals in CSF (eFigure 3A in Supplement 1). However, when comparing metal levels of patients of the implant group with elevated titanium, niobium, and zirconium in serum with matched control participants, significantly higher CSF levels of titanium (implant: 0.75 [0.12-1.40] μg/L; control: 0.57 [0.13-1.10] μg/L; *P* = .04), niobium (implant: 0.02 [0.01-0.16] μg/L; control: 0.01 [0.01-0.03] μg/L; *P* = .04), and zirconium (implant: 0.05 [0.01-0.44] μg/L; control: 0.04 [0.01-0.28] μg/L; *P* = .01) were evident in the arthroprosthetically exposed (eFigure 3B in Supplement 1). The 90% quantile of metal levels in WB and serum of the control group and the median CSF metal levels with ranges of all participants are depicted in eTable 11 in Supplement 1.

**Figure 4.**
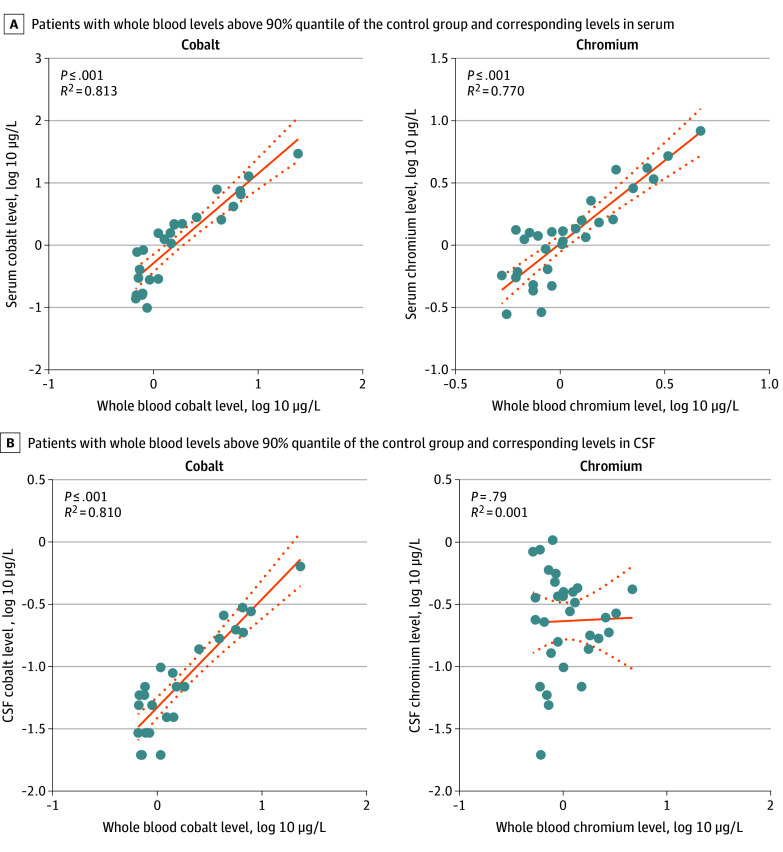
Intermatrix Correlation Analyses and Linear Regression of Log-Transformed Metal Levels of Patients in the Implant Group With Elevated Whole Blood Metal Concentrations The solid lines indicate the linear regression, and dotted lines indicate the 95% confidence bands. A, A total of 26 patients and 31 patients had whole blood cobalt and chromium levels, respectively, greater than the 90% quantile of the control group. B, A total of 41 patients and 38 patients had serum cobalt and chromium levels, respectively, greater than the 90% quantile of the control group.

To analyze whether elevated metal levels in the blood circulation were associated with increased metal levels in CSF, intermatrix correlation analyses of log-transformed metal levels of patients of the implant group were performed. Only patients whose levels of the respective metal were detected as elevated in WB or serum (levels greater than the 90% quantile of the control group) were included in these analyses. Serum and WB levels of cobalt and chromium (Figure 4A) as well as niobium and zirconium (eFigure 4A in Supplement 1) were found to be significantly correlated. Notably, cobalt levels in CSF were significantly correlated with cobalt levels in WB (*r* = 0.82; 95% CI, 0.62-0.92) (Figure 3B) and cobalt levels in serum (*r* = 0.72; 95% CI, 0.53-0.85) (eFigure 4B in Supplement 1). These correlations of log-transformed cobalt values can be considered linear with coefficients of determination of 0.810 and 0.779, respectively. CSF levels of niobium were significantly correlated with WB levels of niobium with a coefficient of determination of 0.160 (eFigure 4C in Supplement 1).

Since distinct exposure to zirconium in the bloodstream was detected in patients with at least 1 arthroplasty implant in situ, and bone cement might be the predominant source of this exposure, it was investigated whether patients with cemented components also exhibited elevated zirconium levels in the CSF. Patients with at least 1 cemented arthroplasty implant component had significantly higher zirconium levels in WB and serum than matched control participants, but not in CSF (eFigure 5 in Supplement 1).

S-100B was quantified as a marker of epithelial barrier integrity in serum to exclude that an impaired blood-CNS barrier integrity contributed to elevated metal levels in CSF. These analyses showed that patients in the implant group had significantly lower serum S-100B levels than matched control participants, and that serum S-100B levels were not age-correlated in either group (eFigure 6 in Supplement 1). To analyze whether lower S-100B levels could be associated with metal exposure in the CNS, S-100B levels of patients with elevated CSF metal levels in the implant group were compared with S-100B levels of matched controls. Patients in the implant group with elevated CSF cobalt levels had significantly lower S-100B levels compared with control participants (eFigure 7A in Supplement 1), whereas S-100B levels were unaffected in patients with nonelevated CSF cobalt levels. Lower serum S-100B levels were not detected in patients in the implant group with elevated CSF chromium levels (eFigure 7B in Supplement 1). Significantly lower S-100B levels were detected in serum of patients with elevated zirconium levels in CSF compared with matched controls, while S-100B levels remained unaffected in patients with nonelevated CSF zirconium levels (eFigure 7C in Supplement 1).

## Discussion

The aims of this study were to investigate whether patients with large artificial joints exhibited elevated levels of metals released from these implants in the blood stream and in CSF. Multimetal quantifications in WB, serum, and CSF revealed that patients with arthroplasty implants were systemically exposed to various arthroplasty metals, including cobalt, chromium, titanium, niobium, zirconium, tantalum, nickel, and vanadium. Subdivision of the implant group and correlation analyses implied arthroplasty implants to be the source of such exposures. In fact, chronic exposure to arthroprosthetic wear and corrosion products was found to be associated with metal accumulation in CSF, but only CSF cobalt levels were significantly higher in patients with joint replacements than in arthroplasty-naive controls. Notably, in the CSF of patients with at least 1 implant component made of cobalt-chromium-molybdenum, cobalt and chromium were found to be significantly higher than in the matched control participants. This finding emphasizes that the use of the cobalt-chromium-molybdenum alloy in particular bears the risk of exposure to arthroprosthetic metals in the CNS.

Previous studies on arthroplasty-associated systemic metal exposure largely focused on cobalt and chromium; some included titanium. Our results suggest that niobium, zirconium, tantalum, nickel, and vanadium levels should also be assessed, and the potential toxic effects of all arthroplasty metals per se and in combination should be considered in new-onset dysfunction as well of periprosthetic tissues (eg, osteolyses) as of any other organ system, with emphasis on the CNS. Patients with elevated titanium, niobium, and zirconium levels in serum also exhibited significantly higher levels of those metals in the CSF. Of note, aluminum was not found to be significantly elevated in any of the examined compartments of patients included, even though aluminum is remarkably prevalent in arthroplasty as a constituent of titanium-aluminum-vanadium and titanium-aluminum-niobium alloys, which are widely used for nonarticulating implant components, and in the form of ceramic composite materials based on aluminum oxide, which are increasingly used as articulating surfaces. Further research should investigate whether this is due to confined release, periprosthetic deposition,^31^ continuous clearance, a combination thereof, or other causes, especially considering persistent concern regarding the potential role of aluminum in neurodegenerative disorders.^32,33^

To date, only one other study^34^ investigating CSF levels of arthroplasty metals has been published. Harrison-Brown et al^34^ exclusively looked at cobalt and chromium levels and compared such in blood plasma and CSF of patients with MoM hip replacements and respective arthroplasty-naive control participants. They detected exchange of cobalt and chromium from plasma to CSF and reported that in patients with MoM implants, the penetration of cobalt in the CSF was limited to 15% of plasma cobalt levels. They further noticed a nonlinear trend with a ceiling effect in the CSF cobalt accumulation in relation to cobalt plasma levels in blood, suggesting a barrier function of the choroid plexus. In our study, we also found that cobalt, rather than chromium, correlated with respective blood levels, although we did not observe a ceiling effect or a nonlinear trend. It is unlikely that this conflicting finding is due to different maximum cobalt levels, because the highest quantified cobalt level in serum in our study was 29.0 μg/L, while Harrison-Brown et al^34^ detected a maximum cobalt level in plasma of 546 nmol/L (32.2 μg/L).^34^ In a case study, massive cobalt and chromium releases from a hip arthroplasty resulting in a WB cobalt level of 549 μg/L and a CSF cobalt level of 11.4 μg/L were reported.^16^ Applying the linear relationship identified in our study between WB and CSF cobalt levels, the CSF cobalt level quantified can be predicted with high precision at 10.8 μg/L. Therefore, we assume that CSF cobalt levels can be predicted based on a linear correlation applying the WB level even in cases of massive cobalt exposure. This is most likely due to the expression of the divalent metal transporter 1 (DMT1), which is also present in blood-CNS barriers with high cobalt ion substrate selectivity.^35,36,37^

The elevated metal levels detected in our study can be attributed to the release from arthroplasty implants, as shown by the comparison of metal levels with arthroplasty-naive patients and also by the significant correlation between cobalt and chromium, the main components of cobalt-chromium-molybdenum alloys. A correlation between cobalt and chromium levels in serum has been demonstrated in previous studies on systemic metal levels in patients with MoM implants.^38,39^ Furthermore, our study revealed that upon comparing cobalt and chromium levels in patients with at least 1 cobalt-chromium-molybdenum–containing implant component, the cobalt and chromium levels in WB, serum, and CSF were significantly higher compared with control participants. The higher chromium levels in CSF are surprising given that chromium levels in serum and WB do not correlate with those in CSF. Hence, higher chromium levels in serum and WB do not necessarily result in increased CSF chromium levels. Yet, patients with cobalt-chromium-molybdenum components still exhibited elevated CSF chromium levels compared with matched control participants. To our knowledge, a specific transport mechanism for chromium through blood-CNS barriers has not been demonstrated. In vitro data suggest that binding of chromium(III) to transferrin does not lead to specific cellular transport.^40^

Titanium, niobium, and zirconium levels were found to be elevated in CSF if these metals were also elevated in serum. As for chromium, levels of these metals did not correlate between serum and CSF. Besides the absence of specific transport mechanisms for these nonessential metals, this could be due to the chemical speciation of the arthroprosthetic degradation products released into circulation. Titanium in the form of titanium dioxide particles has been detected in periprosthetic tissues.^41^ Elevated systemic zirconium levels in our study can primarily be explained by the release of zirconium dioxide (ZrO_2_) from bone cement, as patients with cemented components show significantly higher zirconium levels in WB and serum than controls. ZrO_2_ is used for radiopacity in bone cement, which consists of polymethylmethacrylate (PMMA). Histological examination of periprosthetic tissue showed ZrO_2_ particles embedded in PMMA.^42^ The chemical speciation and physicochemical properties of zirconium, titanium, and niobium in the bloodstream after release from arthroplasty implants and how these parameters influence their transport through blood-CNS barriers are unknown.

A pathologically altered permeability of the blood-CNS barriers in individual patients could additionally explain why arthroplasty metals whose blood and CSF levels do not correlate are significantly elevated in CSF. An elevated S-100B level in serum is considered a clinical parameter of blood-CNS barrier permeability and CNS injury.^43^ To exclude potential influence of reduced blood-CNS barrier function, S-100B levels were quantified. Compared with control participants, we detected lower S-100B levels in the implant group. Thus, it appears unlikely that compromised barrier functions caused the increased metal levels detected in CSF. Lower S-100B levels might be related to metal exposure given that we detected significantly lower S-100B levels in serum of patients with elevated cobalt and zirconium levels in CSF compared with controls. Differences in serum S-100B might be due to slightly higher age of the implant group. However, in our cohort, age and serum S-100B did not correlate. As shown for calcium, copper, and zinc, the binding of divalent metal ions to S-100B might influence S-100B levels in circulation.^44,45^ Still, whether an affinity applies to divalent cobalt and whether the potential binding leads to lower S-100B values in metal-exposed patients remains speculative.

### Strengths and Limitations

Strengths of our study included quantification of all metal constituents of alloys commonly used in arthroplasty implants; adherence to specimen processing in an accredited laboratory, including performance of 3 measurements of each metal quantification; as well as broad eligibility criteria and recruitment regardless of arthroplasty implant performance, design, and survival as well as patients’ comorbidities, making the results generalizable. Limitations included that patients in the implant group were significantly older than control participants, which could result in potential bias since loss in functionality of the blood-CNS barrier with age may create opportunities for metal accumulation in the CNS of older patients. Correlation analyses between age and quantitative metal levels revealed that molybdenum levels in WB were positively correlated with age, while vanadium levels in serum were negatively correlated. This should be considered when interpreting the results of this pilot study. In future prospective studies, age should be considered as a noninterest covariate to control for its potential confounding effects. This pilot study, focusing on investigating the relationship between the presence of arthroplasty implants and systemic metal levels, has a distinctly exploratory nature. The results presented here are based on a cross-sectional observational design and do thus not allow for a clear differentiation between cause and effect but do reveal important associations. A more comprehensive understanding of the findings and potential health consequences of arthroprosthetic metal exposure in the CNS should be pursued in large-scale studies with prospective follow-up. Another limitation is that the study protocol did not include the quantification of further clinically established blood-brain barrier integrity markers (eg, CSF and serum albumin and free light chain) and that neither condition-specific blood-CNS barrier integrity nor individual metal excretion capacity were evaluated, as we recruited participants with a range of diseases, many without validated impact on these parameters. Similarly, we did not exclude individuals who were occupationally exposed to metals, and we cannot rule out with absolute certainty that individuals with an environmental metal exposure they themselves had been unaware of were included in our analyses. On a related note, we would like to highlight that the NeuroWear study was primarily designed as a cross-sectional pilot study analyzing arthroprosthetic metal exposure in 3 biological matrices at a single point of time. As such, it is prone to residual confounding and unsuitable for investigating possible causalities between laboratory data and clinical features. For subsequent studies, a prospective randomized clinical trial design, a much larger sample size, a narrower age difference between study and control groups, and the inclusion of markers of neuronal loss or integrity (eg, serum neurofilament light chain and glial fibrillary acidic protein) that allow for the testing of associations between metals and CNS damage would be advisable. Nonetheless, the pragmatic design reflected a heterogeneous, aged population presenting common disorders of various organ systems in both the implant and control groups, making the results useful to clinicians and patients.

## Conclusions

In conclusion, we showed that cobalt, chromium, titanium, niobium, and zirconium released from arthroplasty implants may not only cross the blood-CNS barriers but may also accumulate in CSF. Considering the findings of this pilot study’s exposure analyses, subsequent studies are needed to determine whether CSF metal concentrations correlate with objective measures of neurotoxic effects. In view of the known neurotoxic potential of especially cobalt, but also of titanium and niobium, this may be particularly relevant in patients with new-onset or deterioration of preexisting neurological or psychiatric disorders following arthroplasty, and in general, in manufacturing orthopedic implants of the future.

## Notes

Supplement 1. **eAppendix 1.** Inclusion and Exclusion Criteria **eAppendix 2.** Procedures **eAppendix 3.** Case Report Form DRKS00014555 **eAppendix 4.** Case Report Form DRKS00014556 **eTable 1.** Matched Study Participants in the Implant and Control Groups **eTable 2.** Enrolled Patients Whose Sample Sets Were Incomplete or Could Not Be Analyzed **eTable 3.** Spearman *r* and *P* Values of Correlation Analyses Between Age and Metal Levels in Whole Blood, Serum and Cerebrospinal Fluid **eTable 4.** Metal Levels [µg/L] Quantified by Inductively Coupled Plasma Mass Spectrometry in Whole Blood of Patients With at Least 1 Arthroplasty Implant in Situ and of Age- and Sex-Matched Arthroplasty Implant-Naive Patients **eTable 5.** Metal Levels Quantified by Inductively Coupled Plasma Mass Spectrometry in Serum of Patients With at Least 1 Arthroplasty Implant in Situ and of Age- and Sex-Matched Arthroplasty Implant–Naive Patients **eTable 6.** Metal levels Quantified by Inductively Coupled Plasma Mass Spectrometry in Cerebrospinal Fluid of Patients With at Least 1 Arthroplasty Implant in Situ and of Age- and Sex-Matched Arthroplasty Implant–Naive Patients **eTable 7.** Median and Maximum Metal Levels of 102 Patients With Arthroplasty Implant, 68 Patients With at Least 1 Cobalt-Chromium-Molybdenum Component, 28 Patients With Arthroplasty Implant Without a Cobalt-Chromium-Molybdenum Component, and Matched Controls **eTable 8.** *P* Values Resulting From the Spearman Correlation of Metal Levels in Whole Blood of Patients With at Least 1 Arthroplasty Implant in Situ **eTable 9.** *P* Values Resulting From the Spearman Correlation of Metal Levels in Serum of Patients With at Least 1 Arthroplasty Implant in Situ **eTable 10.** *P* Values Resulting From the Spearman Correlation of Metal Levels in Cerebrospinal Fluid of Patients With at Least 1 Arthroplasty Implant in Situ **eTable 11.** 90% Quantile (Q0.90) of Metal Levels in Whole Blood and Serum of the Control Group and CSF Metal Levels With Ranges of Patients With Elevated Blood Metals (≥Q0.90) and With at Least 1 Arthroplasty Implant in Situ **eFigure 1.** Cobalt and Chromium Levels of Patients With at Least 1 Cobalt-Chromium-Molybdenum Component and of Matched Control Participants **eFigure 2.** Multimetal Quantification in Whole Blood, Serum, and CSF of Patients With Different Index Arthroplasty Implants **eFigure 3.** Metal Levels of Patients in the Implant Group With Elevated Metals in Whole Blood and/or Serum and the Corresponding Levels in CSF **eFigure 4.** Intermatrix Correlation Analyses and Linear Regression of Log-Transformed Metal Levels of Patients in the Implant Group and Elevated Blood Metals **eFigure 5.** Zirconium Levels in Whole Blood, Serum, and CSF of 62 Patients With at Least 1 Cemented Arthroplasty Implant in Situ, 34 Patients With at Least 1 Arthroplasty Implant but No Bone Cement in Situ, and Matched Controls **eFigure 6.** Serum S-100B Levels of (a) 102 Arthroplasty Implant–Naive Patients and 99 Patients With at Least 1 Arthroplasty Implant in Situ **eFigure 7.** Stratification of Patients According to Implant Status and Quantification of the Blood-CNS Barrier Integrity Marker S-100B

Supplement 2.
Data Sharing Statement
