# Supplementary material for: Metal Concentrations in Blood and Cerebrospinal Fluid of Patients With Arthroplasty Implants
Source: JAMA Netw Open. 2025 Mar 28;8(3):e252281. doi: 10.1001/jamanetworkopen.2025.2281 (PMC11953760; doi:10.1001/jamanetworkopen.2025.2281)
Supplement: Supplement 1. — eAppendix 1. Inclusion and Exclusion Criteria eAppendix 2. Procedures eAppendix 3. Case Report Form DRKS00014555 eAppendix 4. Case Report Form DRKS00014556 eTable 1. Matched Study Participants in the Implant and Control Groups eTable 2. Enrolled Patients Whose Sample Sets Were Incomplete or Could Not Be Analyzed eTable 3. Spearman r and P Values of Correlation Analyses Between Age and Metal Levels in Whole Blood, Serum and Cerebrospinal Fluid eTable 4. Metal Levels [µg/L] Quantified by Inductively Coupled Plasma Mass Spectrometry in Whole Blood of Patients With at Least 1 Arthroplasty Implant in Situ and of Age- and Sex-Matched Arthroplasty Implant-Naive Patients eTable 5. Metal Levels Quantified by Inductively Coupled Plasma Mass Spectrometry in Serum of Patients With at Least 1 Arthroplasty Implant in Situ and of Age- and Sex-Matched Arthroplasty Implant–Naive Patients eTable 6. Metal levels Quantified by Inductively Coupled Plasma Mass Spectrometry in Cerebrospinal Fluid of Patients With at Least 1 Arthroplasty Implant in Situ and of Age- and Sex-Matched Arthroplasty Implant–Naive Patients eTable 7. Median and Maximum Metal Levels of 102 Patients With Arthroplasty Implant, 68 Patients With at Least 1 Cobalt-Chromium-Molybdenum Component, 28 Patients With Arthroplasty Implant Without a Cobalt-Chromium-Molybdenum Component, and Matched Controls eTable 8. P Values Resulting From the Spearman Correlation of Metal Levels in Whole Blood of Patients With at Least 1 Arthroplasty Implant in Situ eTable 9. P Values Resulting From the Spearman Correlation of Metal Levels in Serum of Patients With at Least 1 Arthroplasty Implant in Situ eTable 10. P Values Resulting From the Spearman Correlation of Metal Levels in Cerebrospinal Fluid of Patients With at Least 1 Arthroplasty Implant in Situ eTable 11. 90% Quantile (Q0.90) of Metal Levels in Whole Blood and Serum of the Control Group and CSF Metal Levels With Ranges of Patients With Elevated Blood Metals (≥Q0.90) and With at L [file jamanetwopen-e252281-s001.pdf]

Supplemental Online Content

Rakow A, Kowski A, Treskatsch S, et al. Metal concentrations in CSF, whole blood, and serum of patients with arthroplasty implants. *JAMA Netw Open.* 2025;8(3):e252281. doi:10.1001/jamanetworkopen.2025.2281

eAppendix 1. Inclusion and Exclusion Criteria

eAppendix 2. Procedures

eAppendix 3. Case Report Form DRKS00014555

eAppendix 4. Case Report Form DRKS00014556

eTable 1. Matched Study Participants in the Implant and Control Groups

eTable 2. Enrolled Patients Whose Sample Sets Were Incomplete or Could Not Be Analyzed

eTable 3. Spearman *r* and *P* Values of Correlation Analyses Between Age and Metal Levels in Whole Blood, Serum and Cerebrospinal Fluid

eTable 4. Metal Levels [µg/L] Quantified by Inductively Coupled Plasma Mass Spectrometry in Whole Blood of Patients With at Least 1 Arthroplasty Implant in Situ and of Age- and Sex-Matched Arthroplasty Implant-Naive Patients

eTable 5. Metal Levels Quantified by Inductively Coupled Plasma Mass Spectrometry in Serum of Patients With at Least 1 Arthroplasty Implant in Situ and of Age- and Sex-Matched Arthroplasty Implant–Naive Patients

eTable 6. Metal levels Quantified by Inductively Coupled Plasma Mass Spectrometry in Cerebrospinal Fluid of Patients With at Least 1 Arthroplasty Implant in Situ and of Age- and Sex-Matched Arthroplasty Implant–Naive Patients

eTable 7. Median and Maximum Metal Levels of 102 Patients With Arthroplasty Implant, 68 Patients With at Least 1 Cobalt-Chromium-Molybdenum Component, 28 Patients With Arthroplasty Implant Without a Cobalt-Chromium-Molybdenum Component, and Matched Controls

eTable 8. *P* Values Resulting From the Spearman Correlation of Metal Levels in Whole Blood of Patients With at Least 1 Arthroplasty Implant in Situ

eTable 9. *P* Values Resulting From the Spearman Correlation of Metal Levels in Serum of Patients With at Least 1 Arthroplasty Implant in Situ

eTable 10. *P* Values Resulting From the Spearman Correlation of Metal Levels in Cerebrospinal Fluid of Patients With at Least 1 Arthroplasty Implant in Situ

eTable 11. 90% Quantile (Q0.90) of Metal Levels in Whole Blood and Serum of the Control Group and CSF Metal Levels With Ranges of Patients With Elevated Blood Metals (≥Q0.90) and With at Least 1 Arthroplasty Implant in Situ

eFigure 1. Cobalt and Chromium Levels of Patients With at Least 1 Cobalt-Chromium-Molybdenum Component and of Matched Control Participants

eFigure 2. Multimetal Quantification in Whole Blood, Serum, and CSF of Patients With Different Index Arthroplasty Implants

eFigure 3. Metal Levels of Patients in the Implant Group With Elevated Metals in Whole

© 2025 Rakow A et al. *JAMA Network Open*.

Blood and/or Serum and the Corresponding Levels in CSF

eFigure 4. Intermatrix Correlation Analyses and Linear Regression of Log-Transformed Metal Levels of Patients in the Implant Group and Elevated Blood Metals

eFigure 5. Zirconium Levels in Whole Blood, Serum, and CSF of 62 Patients With at Least 1 Cemented Arthroplasty Implant in Situ, 34 Patients With at Least 1 Arthroplasty Implant but No Bone Cement in Situ, and Matched Controls

eFigure 6. Serum S-100B Levels of (a) 102 Arthroplasty Implant–Naive Patients and 99 Patients With at Least 1 Arthroplasty Implant in Situ

eFigure 7. Stratification of Patients According to Implant Status and Quantification of the Blood-CNS Barrier Integrity Marker S-100B

This supplemental material has been provided by the authors to give readers additional information about their work.

© 2025 Rakow A et al. *JAMA Network Open*.

eAppendix 1. Inclusion and Exclusion Criteria

(1) Inclusion criteria:
Sex: All
Minimum Age: 18 Years
Maximum Age: no maximum age
General Inclusion Criteria:

- written informed consent
- denial of concurrent participation in interventional trial(s) affecting the German Drug Act ("AMG" / "Arzneimittelgesetz")
- only for the study group, not for the control group:
  at least one arthroplasty implant of the hip, knee, ankle, shoulder or elbow joint in situ
- only for the control group:
  no arthroplasty implant of the hip, knee, ankle, shoulder or elbow joint in situ; matched on self-reported sex and age (aiming at the minimal age difference between the two groups, i.e. a mean age difference of ≤ 5 years) with the study group

Additional Inclusion Criteria:
DRKS00014555: scheduled for elective surgery under spinal anesthesia
DRKS00014556: scheduled for lumbar puncture in the course of clinical routine
(i.e. to rule out, detect, monitor, or treat neurological diseases / disorders)

(2) Exclusion criteria:

- no written informed consent
- incapability to give informed consent
- age < 18 years
- concurrent participation in interventional trial(s) affecting the German Drug Act (AMG / "Arzneimittelgesetz")
- prevalence of contraindications of lumbar puncture or spinal anesthesia
- symptomatic heart failure NYHA ≥ III
- symptomatic heart valve disease ≥ II°
- hepatic insufficiency CHILD ≥ B
- acute or chronic renal failure necessitating dialysis
- sepsis
- history of current or previous alcohol or other drug abuse, excl. nicotine / tobacco products

© 2025 Rakow A et al. *JAMA Network Open*.

eAppendix 2. Procedures

We performed multi-metal quantifications in CSF, whole blood (WB) and serum of all participants. Therefore, a CSF sample of 0.60 - 7.50 ml was collected during routine SPA insertion for elective surgery, or a CSF sample of 0.50 - 12.75 ml was collected in the course of routine diagnostic and/or therapeutic LP. WB (>2 ml) and serum (>0.95 ml) were also sampled in the course of routine preoperative / pre-LP workup. SPA, LPs as well as peripheral venous punctures were performed according to established standard operating procedures, including the use of standard cannulas and trace element free vacutainer tubes. The needles for blood and CSF collection were previously tested for any release of metals relevant to this study to exclude contaminations. For multi-metal quantification, WB was sent directly to the laboratory accredited for multi-metal quantification. Serum and CSF tubes were stored at 4°C immediately after collection for a maximum of 24h. Serum was separated by centrifugation at 2000 x g for 10 min. Both, serum and CSF were aliquoted and frozen at -80°C in sterile 2.0 ml screw cap tubes until multi-metal quantification.

Metal ion concentrations were determined by inductively coupled plasma-mass spectrometry (ICP-MS), and all laboratory investigators and staff were blinded to group allocation. In preparation for ICP-MS analysis, EDTA-WB samples were diluted 1:20 in high purity 0.1% NH_3 (Suprapur, Supelco) / 0.02% Lutrol F88 (AppliChem). Serum and CSF specimens were diluted 1:20 in 1% HNO_3 (Suprapur, Supelco). Subsequent multielement analyses were performed in collision/reaction cell mode by ICP-MS (ICapQ, Thermo Fisher), using external and internal standard calibration (Elemental Scientific). Results represent means of three measurements each. Serum levels of S-100B protein were analyzed by an automated CLIA assay (Liaison s100, Diasorin) according to the manufacturer's instructions.

A standardized case report form (supplementary information 3) was used to explore participant's basic demographics, concomitant diseases and medication, orthopedic history, arthroplasty implant data, and individual metal exposure. Data collected for this trial included CRF information, results of metal concentration analyses of CSF, WB and serum, sample volumes and histories, characteristics of devices used to collect CSF, and additional relevant data from electronic medical charts.

© 2025 Rakow A et al. *JAMA Network Open*.

eAppendix 3. Case Report Form DRKS00014555

Center for Musculoskeletal Surgery
Charité – Universitätsmedizin Berlin

Assessment of metal concentrations in cerebrospinal fluid, whole blood and serum of patients with arthroplasty implants in situ – a pilot study

The NeuroWear Study (I)

CASE REPORT FORM

Participant number:

Group allocation: study group / control group

Scheduled surgical procedure:

Indication for surgery:

Date of surgery / spinal anaesthesia,
i.e. date of CSF / whole blood / serum sampling**:**

A) Basic demographic data

Age:

Self-reported sex:

Body height (cm):

Body weight (kg):

© 2025 Rakow A et al. *JAMA Network Open*.

B) Arthropasty history

(If you are a participant of the control group, i.e. if you have not yet undergone replacement of a hip, knee, ankle, shoulder or elbow joint, please proceed to Part C.)

If possible, please provide a copy of your arthroplasty passport.

Which of your joints has/have been replaced by an arthroplasty implant? When did you undergo this / these joint replacement/s ?

1. Date__.____.______
2. Date__.____.______
3. Date__.____.______
4. Date__.____.______
5. Date__.____.______

Have any of the abovementioned arthroplasty implants been revised, i.e. partially or completely exchanged, since primary implantation ?

1. Yes □ No □
2. Yes □ No □
3. Yes □ No □
4. Yes □ No □
5. Yes □ No □

How often have you undergone exchange of the abovementioned arthroplasty implants or components thereof ?

1. 1x □ 2x□ 3x □ 4x□
2. 1x □ 2x□ 3x □ 4x□
3. 1x □ 2x□ 3x □ 4x□
4. 1x □ 2x□ 3x □ 4x□
5. 1x □ 2x□ 3x □ 4x□

© 2025 Rakow A et al. *JAMA Network Open*.

Please indicate the type of your current arthroplasty implant/s:

1.

2.

3.

4.

5.

Please indicate your past arthroplasty implant/s and the respective survival time, i.e. the time span you lived with the specific implant in your body.

1.

2.

3.

4.

5.

Please rate your overall satisfaction with your joint replacement/s (1= very satisfied, 2= satisfied, 3= neutral, 4= dissatisfied, 5 = very dissatisfied):

1. 1 □ 2□ 3 □ 4□ 5□

2. 1 □ 2□ 3 □ 4□ 5□

3. 1 □ 2□ 3 □ 4□ 5□

4. 1 □ 2□ 3 □ 4□ 5□

5. 1 □ 2□ 3 □ 4□ 5□

Have you experienced pain of your replaced hip / knee / ankle / shoulder / elbow joint/s within the past three months?
If yes, please rate your average pain level related to the respected joint on a scale of 1 to 10, with 1 being "no pain" and 10 being "the worst pain you can imagine", at rest versus in motion & under load.

1. Yes □ No □

At rest:

1□ 2□ 3 □ 4□ 5□ 6 □ 7□ 8 □ 9□ 10□

In motion & under load:

1□ 2□ 3 □ 4□ 5□ 6 □ 7□ 8 □ 9□ 10□

© 2025 Rakow A et al. *JAMA Network Open*.

2. Yes ☐ No ☐

At rest:

1☐ 2☐ 3 ☐ 4☐ 5☐ 6 ☐ 7☐ 8 ☐ 9☐ 10☐

In motion & under load:

1☐ 2☐ 3 ☐ 4☐ 5☐ 6 ☐ 7☐ 8 ☐ 9☐ 10☐

3. Yes ☐ No ☐

At rest:

1☐ 2☐ 3 ☐ 4☐ 5☐ 6 ☐ 7☐ 8 ☐ 9☐ 10☐

In motion & under load:

1☐ 2☐ 3 ☐ 4☐ 5☐ 6 ☐ 7☐ 8 ☐ 9☐ 10☐

4. Yes ☐ No ☐

At rest:

1☐ 2☐ 3 ☐ 4☐ 5☐ 6 ☐ 7☐ 8 ☐ 9☐ 10☐

In motion & under load:

1☐ 2☐ 3 ☐ 4☐ 5☐ 6 ☐ 7☐ 8 ☐ 9☐ 10☐

5. Yes ☐ No ☐

At rest:

1☐ 2☐ 3 ☐ 4☐ 5☐ 6 ☐ 7☐ 8 ☐ 9☐ 10☐

In motion & under load:

1☐ 2☐ 3 ☐ 4☐ 5☐ 6 ☐ 7☐ 8 ☐ 9☐ 10☐

© 2025 Rakow A et al. *JAMA Network Open*.

C) Further study-relevant information:

1) Have you had allergic contact dermatitis (i.e. an allergic skin reaction after contact with costume jewelry, belt buckles, textiles, plastics, leather, plasters or similar) in the past ?

Yes ◻ No ◻

If yes, please specify:

2) Are you allergic to metals or have you previously shown a hypersensitivity reaction to metals (e.g. cobalt, chromium, nickel) ?

Yes ◻ No ◻

If yes, please specify:

3) Do you have or have you had frequent contact with metals in the past? (e.g. through working in a metalworking profession)

Yes ◻ No ◻

If yes, please specify (including duration and intensity of exposure, if possible):

4) Do you currently wear or have you worn earrings and/or other piercings in the past?

Yes ◻ No ◻

If yes, please specify:

5) Do you have tattoos?

Yes ◻ No ◻

If yes, please specify (including information regarding when and where the tattoo/s was/were made, and what colors were used):

6) Do you smoke or have you smoked (tobacco) in the past?

Yes ◻ No ◻

If yes, please specify:

Current smoker ◻ Former smoker ◻ Never smoked ◻

Pack-years:

© 2025 Rakow A et al. *JAMA Network Open*.

7) Have you had any other implants inserted in addition to joint prostheses (such as heart/brain pacemakers, dental implants, a "hernia mesh", screws, wires and/or plates for treating bone fractures, etc.)?

If yes, please specify what was inserted / implanted and when was it inserted / implanted? If possible, please provide a copy of the respective implant passport/s or equivalent.

Yes ▫ No ▫

If yes, please specify:

1. Date__.____.______

2. Date__.____.______

3. Date__.____.______

4. Date__.____.______

5. Date__.____.______

Further Date__.____.______

8) Concomitant diseases

Please check "Yes" or "No" for each category. If you check "Yes", please specify.

Neurological disorders:

Yes ▫ No ▫

If yes, please specify:

1.

2.

3.

Further:

Psychiatric disorders:

Yes ▫ No ▫

If yes, please specify:

1.

© 2025 Rakow A et al. *JAMA Network Open*.

2.

3.

Further:

Rheumatic disease/s:

Yes □ No □

If yes, please specify:

1.

2.

3.

Further:

Diabetes mellitus (NIDDM, IDDM):

Yes □ No □

If yes, please specify:

Hyperlipidaemia:

Yes □ No □

If yes, please specify:

1.

2.

3.

Further:

Thyroid diseases / dysfunction:

Yes □ No □

If yes, please specify:

1.

2.

3.

Further:

© 2025 Rakow A et al. *JAMA Network Open*.

Cardiovascular diseases:

Yes ▫ No ▫

If yes, please specify:

1.

2.

3.

Further:

Pulmonary diseases:

Yes ▫ No ▫

If yes, please specify:

1.

2.

3.

Further:

Renal diseases:

Yes ▫ No ▫

If yes, please specify:

1.

2.

3.

Further:

Liver / biliary diseases:

Yes ▫ No ▫

If yes, please specify:

1.

2.

3.

Further:

© 2025 Rakow A et al. *JAMA Network Open*.

Known active malignancy:

Yes ▫ No ▫

If yes, please specify:

1.

2.

3.

Further:

History of previous malignancy:

Yes ▫ No ▫

If yes, please specify:

1.

2.

3.

Further:

9) <u>Concomitant medication:</u>

Current medication: Please provide a copy of your current medication plan, including therapy regimen and respective history if possible.

Provided ▫ Not provided ▫

Existing long-term medications in the past: if possible, please provide details of the duration of use and the medication regime.

Provided ▫ Not provided ▫

If you cannot provide a current medication plan, please check "Yes" or "No" for each category as applicable. If you check "Yes", please specify.

Psychotropic drugs

Yes ▫ No ▫

If yes, please specify:

© 2025 Rakow A et al. *JAMA Network Open*.

Antiepileptic drugs

Yes ◻ No ◻

If yes, please specify:

Medications for the treatment of dementia-related diseases:

Yes ◻ No ◻

If yes, please specify:

Medications for the treatment of Parkinson's disease:

Yes ◻ No ◻

If yes, please specify:

Immunosuppressants/immunomodulators:

Yes ◻ No ◻

If yes, please specify:

Other current medication:

Yes ◻ No ◻

If yes, please specify:

© 2025 Rakow A et al. *JAMA Network Open*.

eAppendix 4. Case Report Form DRKS00014556

CHARITÉ

Center for Musculoskeletal Surgery
Charité – Universitätsmedizin Berlin

Assessment of metal concentrations in cerebrospinal fluid, whole blood and serum of patients with arthroplasty implants in situ who undergo lumbar puncture in the course of the diagnostic workup or therapy of neurological disease(s) – a pilot study

The NeuroWear Study (II)

CASE REPORT FORM

Participant number:

Group allocation: study group / control group

Indication for lumbar puncture:

Date of lumbar puncture,
i.e. date of CSF / whole blood / serum sampling**:**

A) Basic demographic data

Age:

Self-reported sex:

Body height (cm):

Body weight (kg):

© 2025 Rakow A et al. *JAMA Network Open*.

B) Arthropasty history

(If you are a participant of the control group, i.e. if you have not yet undergone replacement of a hip, knee, ankle, shoulder or elbow joint, please proceed to Part C.)

If possible, please provide a copy of your arthroplasty passport.

Which of your joints has/have been replaced by an arthroplasty implant? When did you undergo this / these joint replacement/s ?

1. Date__.____.______
2. Date__.____.______
3. Date__.____.______
4. Date__.____.______
5. Date__.____.______

Have any of the abovementioned arthroplasty implants been revised, i.e. partially or completely exchanged, since primary implantation ?

1. Yes □ No □
2. Yes □ No □
3. Yes □ No □
4. Yes □ No □
5. Yes □ No □

How often have you undergone exchange of the abovementioned arthroplasty implants or components thereof ?

1. 1x □ 2x□ 3x □ 4x□
2. 1x □ 2x□ 3x □ 4x□
3. 1x □ 2x□ 3x □ 4x□
4. 1x □ 2x□ 3x □ 4x□
5. 1x □ 2x□ 3x □ 4x□

© 2025 Rakow A et al. *JAMA Network Open*.

Please indicate the type of your current arthroplasty implant/s:

1.

2.

3.

4.

5.

Please indicate your past arthroplasty implant/s and the respective survival time, i.e. the time span you lived with the specific implant in your body.

1.

2.

3.

4.

5.

Please rate your overall satisfaction with your joint replacement/s (1= very satisfied, 2= satisfied, 3= neutral, 4= dissatisfied, 5 = very dissatisfied):

1. 1 □ 2□ 3 □ 4□ 5□

2. 1 □ 2□ 3 □ 4□ 5□

3. 1 □ 2□ 3 □ 4□ 5□

4. 1 □ 2□ 3 □ 4□ 5□

5. 1 □ 2□ 3 □ 4□ 5□

Have you experienced pain of your replaced hip / knee / ankle / shoulder / elbow joint/s within the past three months?
If yes, please rate your average pain level related to the respected joint on a scale of 1 to 10, with 1 being "no pain" and 10 being "the worst pain you can imagine", at rest versus in motion & under load.

1. Yes □ No □

At rest:

1□ 2□ 3 □ 4□ 5□ 6 □ 7□ 8 □ 9□ 10□

In motion & under load:

1□ 2□ 3 □ 4□ 5□ 6 □ 7□ 8 □ 9□ 10□

© 2025 Rakow A et al. *JAMA Network Open*.

2. Yes □ No □

At rest:

1□ 2□ 3 □ 4□ 5□ 6 □ 7□ 8 □ 9□ 10□

In motion & under load:

1□ 2□ 3 □ 4□ 5□ 6 □ 7□ 8 □ 9□ 10□

3. Yes □ No □

At rest:

1□ 2□ 3 □ 4□ 5□ 6 □ 7□ 8 □ 9□ 10□

In motion & under load:

1□ 2□ 3 □ 4□ 5□ 6 □ 7□ 8 □ 9□ 10□

4. Yes □ No □

At rest:

1□ 2□ 3 □ 4□ 5□ 6 □ 7□ 8 □ 9□ 10□

In motion & under load:

1□ 2□ 3 □ 4□ 5□ 6 □ 7□ 8 □ 9□ 10□

5. Yes □ No □

At rest:

1□ 2□ 3 □ 4□ 5□ 6 □ 7□ 8 □ 9□ 10□

In motion & under load:

1□ 2□ 3 □ 4□ 5□ 6 □ 7□ 8 □ 9□ 10□

© 2025 Rakow A et al. *JAMA Network Open*.

C) Further study-relevant information:

1) Have you had allergic contact dermatitis (i.e. an allergic skin reaction after contact with costume jewelry, belt buckles, textiles, plastics, leather, plasters or similar) in the past ?

Yes ▫ No ▫

If yes, please specify:

2) Are you allergic to metals or have you previously shown a hypersensitivity reaction to metals (e.g. cobalt, chromium, nickel) ?

Yes ▫ No ▫

If yes, please specify:

3) Do you have or have you had frequent contact with metals in the past? (e.g. through working in a metalworking profession)

Yes ▫ No ▫

If yes, please specify (including duration and intensity of exposure, if possible):

4) Do you currently wear or have you worn earrings and/or other piercings in the past?

Yes ▫ No ▫

If yes, please specify:

5) Do you have tattoos?

Yes ▫ No ▫

If yes, please specify (including information regarding when and where the tattoo/s was/were made, and what colors were used):

6) Do you smoke or have you smoked (tobacco) in the past?

Yes ▫ No ▫

If yes, please specify:

Current smoker ▫ Former smoker ▫ Never smoked ▫

Pack-years:

© 2025 Rakow A et al. *JAMA Network Open*.

7) Have you had any other implants inserted in addition to joint prostheses (such as heart/brain pacemakers, dental implants, a “hernia mesh”, screws, wires and/or plates for treating bone fractures, etc.)?

If yes, please specify what was inserted / implanted and when was it inserted / implanted? If possible, please provide a copy of the respective implant passport/s or equivalent.

Yes ▫ No ▫

If yes, please specify:

1. Date__.____.______

2. Date__.____.______

3. Date__.____.______

4. Date__.____.______

5. Date__.____.______

Further Date__.____.______

8) Concomitant diseases

Please check “Yes” or “No” for each category. If you check “Yes”, please specify.

Neurological disorders:

Yes ▫ No ▫

If yes, please specify:

1.

2.

3.

Further:

Psychiatric disorders:

Yes ▫ No ▫

If yes, please specify:

1.

© 2025 Rakow A et al. *JAMA Network Open*.

2.

3.

Further:

Rheumatic disease/s:

Yes □ No □

If yes, please specify:

1.

2.

3.

Further:

Diabetes mellitus (NIDDM, IDDM):

Yes □ No □

If yes, please specify:

Hyperlipidaemia:

Yes □ No □

If yes, please specify:

1.

2.

3.

Further:

Thyroid diseases / dysfunction:

Yes □ No □

If yes, please specify:

1.

2.

3.

Further:

© 2025 Rakow A et al. *JAMA Network Open*.

Cardiovascular diseases:

Yes ▫ No ▫

If yes, please specify:

1.

2.

3.

Further:

Pulmonary diseases:

Yes ▫ No ▫

If yes, please specify:

1.

2.

3.

Further:

Renal diseases:

Yes ▫ No ▫

If yes, please specify:

1.

2.

3.

Further:

Liver / biliary diseases:

Yes ▫ No ▫

If yes, please specify:

1.

2.

3.

Further:

© 2025 Rakow A et al. *JAMA Network Open*.

Known active malignancy:

Yes ◻ No ◻

If yes, please specify:

1.

2.

3.

Further:

History of previous malignancy:

Yes ◻ No ◻

If yes, please specify:

1.

2.

3.

Further:

9) <u>Concomitant medication:</u>

Current medication: Please provide a copy of your current medication plan, including therapy regimen and respective history if possible.

Provided ◻ Not provided ◻

Existing long-term medications in the past: if possible, please provide details of the duration of use and the medication regime.

Provided ◻ Not provided ◻

If you cannot provide a current medication plan, please check "Yes" or "No" for each category as applicable. If you check "Yes", please specify.

Psychotropic drugs

Yes ◻ No ◻

If yes, please specify:

© 2025 Rakow A et al. *JAMA Network Open*.

Antiepileptic drugs

Yes ◻ No ◻

If yes, please specify:

Medications for the treatment of dementia-related diseases:

Yes ◻ No ◻

If yes, please specify:

Medications for the treatment of Parkinson's disease:

Yes ◻ No ◻

If yes, please specify:

Immunosuppressants/immunomodulators:

Yes ◻ No ◻

If yes, please specify:

Other current medication:

Yes ◻ No ◻

If yes, please specify:

© 2025 Rakow A et al. *JAMA Network Open*.

eTable 1. Matched Study Participants in the Implant and Control Groups

implant group	self-reported sex	age [years]	control group	self-reported sex	age [years]
patient 1	m	81	patient 1	m	78
patient 2	w	62	patient 2	w	53
patient 3	w	78	patient 3	w	76
patient 4	w	50	patient 4	w	21
patient 5	w	73	patient 5	W	70
patient 6	m	77	patient 6	m	73
patient 7	w	75	patient 7	w	71
patient 8	w	61	patient 8	w	52
patient 9	w	64	patient 9	w	54
patient 10	w	81	patient 10	w	78
patient 11	m	74	patient 11	m	67
patient 12	m	74	patient 12	m	66
patient 13	w	81	patient 13	w	77
patient 14	m	38	patient 14	m	39
patient 15	m	83	patient 15	m	80
patient 16	w	57	patient 16	w	45
patient 17	w	71	patient 17	w	66
patient 18	w	63	patient 18	w	53
patient 19	m	85	patient 19	m	81
patient 20	w	87	patient 20	w	80
patient 21	w	76	patient 21	w	73
patient 22	m	76	patient 22	m	71
patient 23	w	65	patient 23	w	56
patient 24	m	62	patient 24	m	62
patient 25	m	57	patient 25	m	58
patient 26	m	57	patient 26	m	56
patient 27	w	81	patient 27	w	78
patient 28	m	55	patient 28	m	56
patient 29	m	61	patient 29	m	61
patient 30	w	53	patient 30	w	34
patient 31	m	88	patient 31	m	85
patient 32	w	79	patient 32	w	76
patient 33	m	65	patient 33	m	65
patient 34	m	58	patient 34	m	59
patient 35	w	60	patient 35	w	51
patient 36	w	58	patient 36	w	51
patient 37	w	87	patient 37	w	84
patient 38	w	67	patient 38	w	60
patient 39	m	54	patient 39	m	56

© 2025 Rakow A et al. *JAMA Network Open*.

patient 40	m	81	patient 40	m	76
patient 41	m	73	patient 41	m	67
patient 42	w	74	patient 42	w	70
patient 43	w	68	patient 43	w	61
patient 44	m	75	patient 44	m	69
patient 45	w	77	patient 45	w	76
patient 46	m	77	patient 46	m	72
patient 47	m	83	patient 47	m	80
patient 48	w	85	patient 48	w	79
patient 49	w	77	patient 49	w	76
patient 50	w	58	patient 50	w	49
patient 51	m	58	patient 51	m	59
patient 52	w	84	patient 52	w	79
patient 53	w	73	patient 53	w	69
patient 54	w	69	patient 54	w	63
patient 55	m	62	patient 55	m	63
patient 56	m	58	patient 56	m	59
patient 57	w	56	patient 57	w	41
patient 58	w	70	patient 58	w	63
patient 59	w	70	patient 59	w	64
patient 60	w	76	patient 60	w	75
patient 61	m	78	patient 61	m	75
patient 62	w	72	patient 62	w	68
patient 63	w	76	patient 63	w	74
patient 64	m	83	patient 64	m	80
patient 65	m	61	patient 65	m	61
patient 66	m	74	patient 66	m	67
patient 67	m	81	patient 67	m	76
patient 68	m	61	patient 68	m	61
patient 69	m	82	patient 69	m	79
patient 70	m	81	patient 70	m	77
patient 71	w	64	patient 71	w	56
patient 72	w	70	patient 72	w	64
patient 73	m	82	patient 73	m	78
patient 74	w	56	patient 74	w	43
patient 75	w	78	patient 75	w	76
patient 76	m	72	patient 76	m	66
patient 77	m	76	patient 77	m	72
patient 78	w	64	patient 78	w	56
patient 79	w	70	patient 79	w	64
patient 80	w	78	patient 80	w	76

© 2025 Rakow A et al. *JAMA Network Open*.

patient 81	w	80	patient 81	w	69
patient 82	w	85	patient 82	w	79
patient 83	w	75	patient 83	w	73
patient 84	w	88	patient 84	w	93
patient 85	m	56	patient 85	m	56
patient 86	m	75	patient 86	M	70
patient 87	w	77	patient 87	w	75
patient 88	w	72	patient 88	w	68
patient 89	w	71	patient 89	w	64
patient 90	w	77	patient 90	w	75
patient 91	w	81	patient 91	w	78
patient 92	m	82	patient 92	m	78
patient 93	m	87	patient 93	m	82
patient 94	m	53	patient 94	m	53
patient 95	w	85	patient 95	w	79
patient 96	m	82	patient 96	m	79
patient 97	m	63	patient 97	m	64
patient 98	w	65	patient 98	w	59
patient 99	w	70	patient 99	w	64
patient 100	w	82	patient 100	w	78
patient 101	w	54	patient 101	w	40
patient 102	w	73	patient 102	w	69

© 2025 Rakow A et al. *JAMA Network Open*.

eTable 2. Enrolled Patients Whose Sample Sets Were Incomplete or Could Not Be Analyzed

implant group	self-reported sex	age [years]	control group	self-reported sex	age [years]
patient 103	w	56	patient 103	m	56
			patient 104	m	77
			patient 105	m	74
			patient 106	w	80
			patient 107	m	45
			patient 108	w	79

© 2025 Rakow A et al. *JAMA Network Open*.

eTable 3. Spearman *r* and *P* Values of Correlation Analyses Between Age and Metal Levels in Whole Blood, Serum and Cerebrospinal Fluid

The p-value was corrected for multiple comparisons using the Bonferroni method. A p-value of <0.005 was considered statistically significant. Statistically significant p-values are highlighted in bold.

	Co	Cr	Mo	Ni	Ti	Al	V	Nb	Ta	Zr
**Whole blood**										
Spearman r	-0.080	0.067	0.318	0.123	-0.072	0.047	0.035	-0.154	-0.051	0.036
p-value	0.42	0.51	**0.001**	0.22	0.47	0.64	0.73	0.12	0.612	0.72
**Serum**										
Spearman r	0.092	0.046	0.128	-0.091	-0.110	-0.021	-0.312	-0.133	-0.093	-0.046
p-value	0.36	0.64	0.20	0.36	0.27	0.83	**0.001**	0.18	0.351	0.64
**Cerebrospinal fluid**										
Spearman r	0.140	-0.029	0.268	-0.003	-0.063	0.015	0.019	-0.004	-	-0.004
p-value	0.16	0.77	0.01	0.98	0.53	0.88	0.85	0.97	-	0.97

© 2025 Rakow A et al. *JAMA Network Open*.

eTable 4. Metal Levels [µg/L] Quantified by Inductively Coupled Plasma Mass Spectrometry in Whole Blood of Patients With at Least 1 Arthroplasty Implant in Situ and of Age- and Sex-Matched Arthroplasty Implant–Naive Patients

	Co	Cr	Mo	Ni	Ti	Al	V	Nb	Ta	Zr
**implant group**										
patient 1	0.12	0.75	0.70	1.20	5.80	4.02	0.01	0.02	0.01	0.02
patient 2	0.20	0.48	0.50	1.20	9.20	6.80	0.01	0.01	0.01	0.05
patient 3	0.12	0.42	1.60	0.60	10.80	6.26	0.05	0.01	0.59	0.03
patient 4	0.19	0.40	0.70	1.00	9.30	6.41	0.05	0.01	0.01	0.70
patient 5	0.26	0.56	0.40	2.00	4.70	2.94	0.06	0.01	0.01	0.03
patient 6	0.15	0.46	0.60	1.30	5.30	4.76	0.04	0.02	0.03	0.04
patient 7	1.10	0.75	0.50	1.00	8.00	8.50	0.09	0.04	0.01	0.05
patient 8	0.54	0.44	0.30	0.50	9.60	10.65	0.09	0.01	0.01	0.02
patient 9	0.56	0.43	0.60	3.40	8.20	2.03	0.06	0.02	0.01	0.01
patient 10	6.73	3.32	0.90	1.10	9.40	6.52	0.07	0.01	0.01	0.02
patient 11	0.27	0.41	0.70	0.70	9.30	8.49	0.30	0.01	0.01	0.04
patient 12	0.77	0.49	0.50	0.80	5.20	4.12	0.01	0.02	0.04	0.02
patient 13	0.91	0.48	0.70	0.90	13.60	5.59	0.11	0.03	0.01	0.05
patient 14	0.61	0.46	0.70	0.80	6.40	3.91	0.09	0.03	0.01	0.06
patient 15	0.28	0.45	0.50	0.70	10.00	5.15	0.14	0.08	0.01	0.13
patient 16	0.12	0.47	0.30	1.10	8.60	6.10	0.05	0.06	0.01	0.06
patient 17	0.86	0.56	0.50	0.90	9.50	4.88	0.10	0.05	0.02	0.10
patient 18	0.67	0.48	0.70	0.90	9.60	5.88	0.18	0.04	0.01	0.23
patient 19	5.77	2.26	0.60	1.50	18.10	7.48	0.09	0.06	0.01	0.01
patient 20	1.46	0.68	0.70	0.70	10.00	3.92	0.07	0.03	0.01	0.08
patient 21	0.36	0.39	0.70	1.00	9.30	10.50	0.08	0.04	0.02	0.07
patient 22	0.69	0.49	0.40	0.70	7.90	2.84	0.09	0.08	0.05	0.08
patient 23	0.10	0.43	0.30	0.40	4.00	8.66	0.01	0.01	0.01	0.01
patient 24	0.07	0.39	0.70	0.70	1.80	3.11	0.03	0.03	0.01	0.01
patient 25	0.21	0.45	0.50	0.50	7.60	6.66	0.05	0.01	0.01	0.04
patient 26	0.54	0.33	0.30	1.20	5.60	6.50	0.03	0.01	0.01	0.13

© 2025 Rakow A et al. *JAMA Network Open*.

	Co	Cr	Mo	Ni	Ti	Al	V	Nb	Ta	Zr
patient 27	0.68	0.48	0.50	1.60	6.90	5.95	0.04	0.01	0.01	0.49
patient 28	0.26	0.42	0.50	0.80	7.30	9.30	0.01	0.01	0.01	0.19
patient 29	8.10	2.64	0.60	1.50	5.60	5.30	0.04	0.01	0.01	0.55
patient 30	1.26	1.20	0.50	1.20	14.40	4.86	0.04	0.17	0.01	39.90
patient 31	2.57	1.04	0.40	1.20	11.10	3.76	0.03	0.01	0.01	0.35
patient 32	0.78	0.82	0.70	1.70	4.80	11.90	0.02	0.01	0.01	0.22
patient 33	0.71	0.92	0.30	9.70	6.80	8.93	0.12	0.02	0.01	0.02
patient 34	0.19	0.28	0.50	1.20	7.60	2.89	0.03	0.07	0.16	0.01
patient 35	0.30	0.62	0.40	1.20	9.90	7.59	0.05	0.03	0.01	0.07
patient 36	0.65	0.47	0.50	1.10	8.80	4.69	0.05	0.01	0.01	0.01
patient 37	0.14	0.40	0.50	0.80	5.70	9.80	0.03	0.02	0.01	0.06
patient 38	0.24	1.04	0.40	1.60	10.10	8.36	0.01	0.01	0.01	0.42
patient 39	0.10	0.35	0.30	0.40	6.70	4.64	0.02	0.01	0.01	0.02
patient 40	0.23	0.38	0.50	1.00	7.40	5.05	0.06	0.03	0.01	0.04
patient 41	0.24	0.25	0.40	0.50	7.20	5.36	0.03	0.01	0.01	0.03
patient 42	0.15	0.38	0.60	1.10	9.50	1.64	0.04	0.02	0.01	0.01
patient 43	0.09	0.38	0.50	0.80	6.20	2.18	0.07	0.02	0.01	0.01
patient 44	0.35	1.81	0.80	1.90	10.20	8.57	0.06	0.03	0.01	0.16
patient 45	0.26	0.51	0.60	2.30	5.80	9.37	0.01	0.01	0.02	0.01
patient 46	0.15	0.48	0.50	0.90	5.50	9.84	0.06	0.03	0.01	0.02
patient 47	0.31	0.51	0.40	2.30	8.70	9.52	0.09	0.01	0.01	0.02
patient 48	0.36	0.48	0.80	3.70	7.10	1.91	0.03	0.05	0.01	0.05
patient 49	0.21	0.49	0.50	1.60	7.60	8.53	0.04	0.03	0.01	0.01
patient 50	0.16	0.48	0.30	0.60	9.20	10.24	0.08	0.01	0.01	0.34
patient 51	0.34	0.45	0.30	2.70	2.80	3.44	0.03	0.01	0.01	0.01
patient 52	0.13	0.47	0.50	1.70	3.20	2.83	0.02	0.01	0.01	0.12
patient 53	0.12	0.42	0.60	0.80	12.80	9.07	0.01	0.05	0.01	0.01
patient 54	0.12	0.35	0.70	0.50	8.10	3.34	0.04	0.01	0.01	0.03
patient 55	0.15	0.48	0.40	0.90	5.30	8.37	0.13	0.01	0.01	0.44

© 2025 Rakow A et al. *JAMA Network Open*.

	Co	Cr	Mo	Ni	Ti	Al	V	Nb	Ta	Zr
patient 56	0.47	0.25	0.40	0.40	12.70	9.80	0.08	0.01	0.01	0.06
patient 57	0.34	0.72	0.40	1.20	7.50	5.78	0.09	0.01	0.01	0.44
patient 58	0.25	0.63	0.40	0.90	13.70	10.51	0.13	0.11	0.01	0.01
patient 59	0.13	1.03	0.60	1.00	10.40	5.81	0.09	0.02	0.01	0.65
patient 60	0.38	0.47	0.60	6.50	13.20	5.85	0.05	0.01	0.01	0.05
patient 61	4.42	1.28	0.30	0.80	12.50	7.39	0.07	1.14	0.01	0.03
patient 62	0.12	0.35	0.30	0.40	6.60	4.85	0.30	0.01	0.01	0.01
patient 63	0.27	0.42	0.60	0.70	8.10	7.42	0.02	0.01	0.01	0.58
patient 64	0.18	0.47	0.50	1.50	9.60	3.97	0.04	0.01	0.01	0.46
patient 65	1.87	1.34	0.50	1.40	13.50	8.55	0.12	0.05	0.01	1.91
patient 66	1.10	0.46	0.20	0.90	6.60	3.14	0.05	0.16	0.01	0.13
patient 67	0.23	0.50	0.50	1.20	4.10	9.11	0.15	0.01	0.01	0.01
patient 68	1.56	0.62	0.50	0.80	8.10	5.55	0.08	0.01	0.01	1.14
patient 69	0.17	1.42	1.20	5.10	10.50	6.35	0.11	0.22	0.01	0.15
patient 70	4.01	1.87	0.50	8.10	6.90	5.01	0.09	0.03	0.01	1.34
patient 71	0.59	0.29	0.50	0.80	5.90	3.81	0.07	0.01	0.01	0.17
patient 72	0.16	0.37	0.50	5.00	6.10	9.63	0.09	0.03	0.01	0.08
patient 73	0.24	0.27	0.30	17.30	5.00	9.25	0.12	0.01	0.01	0.05
patient 74	0.14	0.46	0.50	0.60	8.00	8.01	0.05	0.01	0.01	0.03
patient 75	0.38	0.50	0.60	1.90	8.20	3.89	0.10	0.01	0.01	0.02
patient 76	0.19	0.39	1.20	3.20	8.20	7.39	0.05	0.02	0.01	0.01
patient 77	0.54	1.55	0.50	5.50	1.14	2.92	0.03	0.01	0.01	0.03
patient 78	0.64	0.53	0.40	0.70	3.60	4.15	0.04	0.01	0.01	0.09
patient 79	0.13	0.44	0.50	0.70	7.30	5.58	0.03	0.06	0.01	0.11
patient 80	24.10	4.76	1.10	0.80	11.50	7.84	0.06	0.04	0.02	0.01
patient 81	0.11	0.32	0.60	0.60	6.90	5.63	0.03	0.04	0.01	0.07
patient 82	0.10	0.45	0.50	1.60	9.60	5.92	0.01	0.03	0.01	0.01
patient 83	0.17	0.34	0.60	0.70	7.10	6.23	0.13	0.06	0.01	0.13
patient 84	1.57	0.86	0.60	0.80	37.20	5.22	0.81	0.12	0.01	0.05

© 2025 Rakow A et al. *JAMA Network Open*.

	Co	Cr	Mo	Ni	Ti	Al	V	Nb	Ta	Zr
patient 85	0.79	0.49	0.50	1.30	7.70	7.55	0.17	0.05	0.20	0.08
patient 86	0.51	0.79	0.20	0.80	4.30	24.12	0.36	0.01	0.01	0.09
patient 87	0.16	0.41	0.30	3.20	8.40	8.72	0.07	0.10	0.19	0.21
patient 88	0.33	0.46	0.60	0.20	3.90	3.99	0.02	0.01	0.01	0.03
patient 89	0.36	0.41	0.50	1.40	8.60	4.61	0.08	0.09	0.03	0.03
patient 90	0.16	0.45	0.30	0.50	3.40	6.31	0.02	0.01	0.01	0.01
patient 91	0.11	0.37	0.60	0.50	7.50	8.40	0.02	0.02	0.01	0.01
patient 92	0.19	0.50	0.50	0.50	12.10	10.72	0.01	0.07	0.01	0.06
patient 93	0.28	0.32	0.90	0.40	8.40	5.38	0.07	0.03	0.05	0.01
patient 94	0.14	0.47	0.60	0.60	9.40	8.37	0.07	0.01	0.02	0.07
patient 95	1.44	0.92	0.50	0.50	5.00	8.36	0.08	0.01	0.03	6.08
patient 96	6.82	2.83	0.40	1.20	11.90	7.53	0.05	0.13	0.03	1.33
patient 97	0.15	0.24	0.30	16.50	4.00	6.65	0.07	0.03	0.03	0.02
patient 98	0.56	0.88	0.70	17.00	10.00	5.87	0.08	0.03	0.02	0.20
patient 99	0.27	0.32	0.60	1.00	7.30	6.78	0.06	0.70	0.01	0.05
patient 100	0.73	0.34	0.30	1.30	11.70	7.27	0.04	0.02	0.01	0.03
patient 101	0.15	0.33	0.50	1.60	12.20	5.00	0.07	0.01	0.01	0.03
patient 102	0.19	0.45	0.60	1.40	9.50	9.67	0.01	0.01	0.03	0.04
**control group**										
patient 1	0.76	0.52	0.80	1.90	9.40	7.99	0.10	0.01	0.01	0.06
patient 2	0.09	0.31	0.70	0.80	7.50	5.59	0.08	0.01	0.02	0.01
patient 3	0.15	0.51	0.30	1.10	12.10	9.54	0.06	0.01	0.01	0.04
patient 4	0.32	0.44	0.80	0.80	7.40	4.70	0.06	0.01	0.01	0.03
patient 5	0.20	0.46	0.40	1.80	6.10	2.69	0.08	0.01	0.01	0.03
patient 6	0.80	0.46	0.70	1.10	5.50	5.21	0.06	0.02	0.01	0.11
patient 7	0.13	0.21	0.50	22.20	6.30	6.52	0.07	0.02	0.01	0.01
patient 8	0.10	0.36	0.30	0.40	5.70	4.87	0.03	0.01	0.01	0.01
patient 9	0.09	0.42	0.40	0.80	10.30	8.28	0.06	0.01	0.01	0.05
patient 10	0.08	0.34	0.40	0.60	7.10	3.45	0.05	0.01	0.02	0.03

© 2025 Rakow A et al. *JAMA Network Open*.

	Co	Cr	Mo	Ni	Ti	Al	V	Nb	Ta	Zr
patient 11	0.08	0.34	0.40	0.60	7.10	8.69	0.09	0.04	0.02	0.03
patient 12	0.10	0.50	0.50	0.60	8.80	7.95	0.05	0.01	0.01	0.01
patient 13	0.66	0.76	0.70	2.10	7.20	6.55	0.12	0.02	0.01	0.01
patient 14	0.17	0.32	0.50	2.10	3.40	2.06	0.05	0.05	0.01	0.03
patient 15	0.13	0.48	0.50	1.30	6.40	4.70	0.04	0.01	0.01	0.02
patient 16	0.61	0.41	0.40	0.80	9.40	9.50	0.01	0.02	0.01	0.07
patient 17	0.11	0.42	0.50	1.00	10.60	11.31	0.01	0.01	0.01	0.01
patient 18	0.51	0.38	0.30	0.70	9.90	6.70	0.07	0.02	0.01	0.01
patient 19	0.13	0.75	1.70	0.90	6.00	5.41	0.14	0.04	0.01	0.09
patient 20	0.99	0.48	0.80	1.30	6.50	6.74	0.04	0.01	0.01	0.01
patient 21	0.17	0.40	0.80	1.00	4.90	3.35	0.11	0.01	0.01	0.01
patient 22	0.10	0.38	0.50	0.60	5.90	4.00	0.10	0.01	0.01	0.01
patient 23	0.69	0.64	0.40	2.10	5.10	4.28	0.05	0.11	0.01	0.04
patient 24	0.18	0.42	0.50	0.60	9.50	5.91	0.08	0.02	0.01	0.02
patient 25	0.24	0.59	0.40	1.40	4.00	6.10	0.12	0.01	0.01	0.01
patient 26	0.52	0.41	0.60	0.70	6.50	3.22	0.07	0.02	0.01	0.04
patient 27	0.18	0.46	0.30	1.50	6.70	9.25	0.04	0.03	0.01	0.02
patient 28	0.15	0.30	0.30	0.40	4.20	6.54	0.07	0.03	0.01	0.01
patient 29	0.11	0.32	0.20	0.80	10.40	3.01	0.13	0.01	0.01	0.01
patient 30	0.79	0.80	0.30	1.50	7.00	5.76	0.09	0.02	0.01	0.07
patient 31	0.13	0.34	0.80	0.90	4.40	5.48	0.04	0.01	0.01	0.01
patient 32	0.24	0.33	0.80	1.00	9.00	9.21	0.09	0.03	0.06	0.08
patient 33	0.14	0.35	0.50	0.50	4.80	6.60	0.07	0.03	0.01	0.01
patient 34	0.13	0.32	0.60	0.40	8.60	8.14	0.06	0.01	0.01	0.01
patient 35	0.14	0.48	0.80	2.20	8.80	7.94	0.06	0.02	0.01	0.01
patient 36	0.16	0.37	0.70	0.60	6.20	3.51	0.07	0.02	0.01	0.02
patient 37	0.26	0.31	0.70	1.60	4.50	4.51	0.09	0.01	0.01	0.01
patient 38	0.13	0.25	0.60	0.50	9.60	6.73	0.04	0.01	0.01	0.01
patient 39	0.16	0.94	0.30	1.10	20.70	65.40	0.01	0.01	0.02	0.31

© 2025 Rakow A et al. *JAMA Network Open*.

	Co	Cr	Mo	Ni	Ti	Al	V	Nb	Ta	Zr
patient 40	0.15	0.30	0.70	0.30	9.50	5.49	0.07	0.08	0.07	0.01
patient 41	0.09	0.31	0.60	0.70	5.00	8.02	0.06	0.01	0.01	0.04
patient 42	0.16	0.48	0.40	1.80	8.90	5.16	0.08	0.02	0.01	0.02
patient 43	0.67	0.44	0.50	0.80	3.20	3.05	0.02	0.01	0.01	0.04
patient 44	0.13	0.34	0.30	0.50	8.00	5.04	0.05	0.01	0.01	0.32
patient 45	0.53	0.42	0.80	0.90	6.90	8.81	0.04	0.01	0.01	0.04
patient 46	0.11	0.28	0.40	1.40	9.80	6.86	0.05	0.01	0.01	0.38
patient 47	0.17	0.33	0.70	0.90	7.40	3.64	0.02	0.01	0.01	0.03
patient 48	0.18	0.51	0.80	0.90	9.00	10.90	0.01	0.01	0.01	0.80
patient 49	0.10	0.34	0.40	0.90	7.40	9.39	0.06	0.02	0.01	0.02
patient 50	0.90	0.42	0.30	1.20	6.00	8.98	0.01	0.01	0.01	0.03
patient 51	0.19	0.41	0.40	0.60	8.20	3.67	0.06	0.01	0.02	0.03
patient 52	0.63	0.38	0.50	0.60	6.10	4.65	0.03	0.01	0.01	1.95
patient 53	0.11	0.35	0.50	0.70	4.70	2.70	0.01	0.10	0.01	0.02
patient 54	0.23	0.34	0.50	0.90	7.80	7.95	0.11	0.01	0.01	0.03
patient 55	0.09	0.44	0.40	0.70	12.90	8.51	0.08	0.01	0.01	0.06
patient 56	0.13	0.39	0.40	1.00	1.80	2.43	0.03	0.01	0.01	0.01
patient 57	0.13	0.45	0.80	2.30	6.60	6.04	0.05	0.11	0.01	0.01
patient 58	0.10	0.37	0.50	0.60	7.30	4.95	0.01	0.01	0.01	0.47
patient 59	0.17	0.46	0.60	0.80	8.40	6.93	0.08	0.03	0.02	0.05
patient 60	0.37	0.48	0.90	1.20	9.80	10.26	0.13	0.02	0.01	0.05
patient 61	0.09	0.42	0.80	1.40	2.60	9.64	0.09	0.01	0.01	0.01
patient 62	0.20	0.46	0.70	10.10	8.80	7.42	0.01	0.02	0.03	0.06
patient 63	0.13	0.48	0.60	1.00	4.80	4.43	0.01	0.01	0.01	0.01
patient 64	0.84	0.64	1.10	1.90	8.10	3.76	0.12	0.01	0.01	0.03
patient 65	0.71	0.40	0.30	0.70	8.40	4.94	0.16	0.01	0.01	0.10
patient 66	0.18	0.31	2.40	0.70	4.60	6.87	0.06	0.01	0.01	0.01
patient 67	0.60	0.47	0.70	1.10	8.50	7.56	0.19	0.01	0.01	0.65
patient 68	0.11	0.38	0.90	0.90	4.30	8.71	0.01	0.01	0.01	0.01

© 2025 Rakow A et al. *JAMA Network Open*.

	Co	Cr	Mo	Ni	Ti	Al	V	Nb	Ta	Zr
patient 69	0.19	0.27	0.50	0.60	6.40	6.53	0.07	0.04	0.01	0.02
patient 70	0.09	0.41	0.40	0.60	2.70	2.86	0.06	0.01	0.01	0.01
patient 71	0.14	0.25	0.30	0.60	6.10	6.00	0.06	0.01	0.01	0.01
patient 72	0.74	0.52	0.40	0.90	6.70	6.80	0.04	0.01	0.01	0.01
patient 73	0.23	0.24	0.70	0.60	8.00	5.40	0.03	0.01	0.01	0.14
patient 74	0.61	0.47	0.80	0.60	9.50	5.78	0.08	0.03	0.01	0.08
patient 75	0.13	0.51	0.80	0.80	7.30	9.10	0.02	0.01	0.01	0.78
patient 76	0.56	0.36	0.70	0.60	3.80	7.95	0.01	0.01	0.01	0.01
patient 77	0.13	0.44	0.40	2.40	11.00	4.97	0.01	0.01	0.01	0.01
patient 78	0.79	0.47	0.70	1.00	15.50	9.05	0.14	0.03	0.01	0.05
patient 79	0.61	0.45	0.40	0.80	7.80	3.98	0.04	0.01	0.01	0.02
patient 80	0.21	1.52	0.80	14.90	14.40	16.32	0.05	0.03	0.01	0.03
patient 81	0.11	0.37	0.40	0.90	11.80	7.01	0.05	0.01	0.01	0.02
patient 82	0.12	0.50	0.30	1.10	5.90	10.20	0.04	0.02	0.01	0.02
patient 83	0.12	0.31	0.40	0.40	7.30	7.01	0.07	0.02	0.03	0.01
patient 84	0.11	0.65	0.70	1.10	7.40	6.39	0.10	0.01	0.01	0.03
patient 85	0.14	0.43	0.30	1.20	4.80	7.44	0.08	0.01	0.01	0.02
patient 86	0.12	0.44	0.60	0.90	6.50	6.32	0.09	0.01	0.01	0.01
patient 87	0.11	0.37	0.60	0.80	4.50	8.16	0.01	0.01	0.01	0.06
patient 88	0.15	0.33	0.90	0.80	9.60	4.36	0.09	0.03	0.01	0.04
patient 89	0.64	0.37	0.80	0.90	5.40	4.33	0.08	0.01	0.01	0.03
patient 90	0.49	0.36	0.40	0.70	6.30	3.87	0.04	0.01	0.01	0.02
patient 91	0.19	0.41	0.80	1.70	6.70	10.14	0.11	0.03	0.01	0.06
patient 92	0.16	0.49	0.60	0.80	14.80	7.62	0.07	0.01	0.01	0.11
patient 93	0.15	0.48	0.80	0.80	9.60	5.71	0.01	0.01	0.01	0.01
patient 94	0.64	0.44	0.50	11.20	11.90	4.53	0.05	0.02	0.01	0.06
patient 95	0.10	0.31	0.70	0.80	4.60	3.46	0.09	0.01	0.01	0.01
patient 96	0.22	0.46	0.30	1.00	5.70	8.64	0.18	0.01	0.01	0.22
patient 97	0.11	0.49	0.60	63.30	4.10	7.08	0.07	0.01	0.01	0.02

© 2025 Rakow A et al. *JAMA Network Open*.

	Co	Cr	Mo	Ni	Ti	Al	V	Nb	Ta	Zr
patient 98	0.22	0.45	0.40	0.70	8.10	7.43	0.14	0.03	0.02	0.06
patient 99	0.13	0.39	0.40	1.50	7.20	2.98	0.01	0.01	0.01	0.01
patient 100	0.23	0.39	0.70	1.00	5.00	3.81	0.06	0.01	0.01	0.01
patient 101	0.60	0.44	0.40	1.00	8.80	6.83	0.07	0.01	0.01	0.06
patient 102	0.11	0.31	0.40	0.50	7.00	8.81	0.02	0.01	0.01	0.04

© 2025 Rakow A et al. *JAMA Network Open*.

eTable 5. Metal Levels [µg/L] Quantified by Inductively Coupled Plasma Mass Spectrometry in Serum of Patients With at Least 1 Arthroplasty Implant in Situ and of Age- and Sex-Matched Arthroplasty Implant–Naive Patients

	Co	Cr	Mo	Ni	Ti	Al	V	Nb	Ta	Zr
**implant group**										
patient 1	0.10	0.43	1.10	0.55	5.83	30.22	3.17	0.02	0.01	0.08
patient 2	0.18	0.27	0.66	0.76	5.00	24.34	3.30	0.01	0.01	0.11
patient 3	0.17	0.23	2.63	0.59	4.14	30.51	4.13	0.01	0.01	0.09
patient 4	0.14	0.24	1.14	0.23	5.44	36.45	3.98	0.01	0.01	16.17
patient 5	0.22	0.28	0.80	0.53	8.45	20.86	4.11	0.14	0.01	0.17
patient 6	0.18	0.37	1.32	0.90	3.95	19.28	4.09	0.01	0.01	0.18
patient 7	0.29	0.48	0.40	0.01	3.68	19.77	2.51	0.06	0.01	0.17
patient 8	0.21	0.37	0.79	0.57	3.89	15.32	4.35	0.01	0.01	0.10
patient 9	0.12	0.31	1.28	0.39	4.91	32.27	4.40	0.07	0.05	0.20
patient 10	7.38	5.14	1.76	2.03	6.79	25.67	3.97	0.06	0.07	0.27
patient 11	0.07	0.37	1.20	0.41	5.07	22.91	3.82	0.01	0.01	0.11
patient 12	0.16	0.28	0.96	0.14	10.03	39.01	4.02	0.12	0.23	0.07
patient 13	0.28	0.44	1.08	0.48	2.82	32.68	3.79	0.01	0.01	0.08
patient 14	0.12	0.31	1.19	0.49	4.76	26.93	3.54	0.03	0.01	0.32
patient 15	0.22	0.26	1.15	0.01	5.09	38.38	4.34	0.01	0.01	0.30
patient 16	0.09	0.34	0.82	0.53	7.66	31.09	4.15	0.03	0.01	0.13
patient 17	0.10	0.28	0.96	0.47	4.01	27.97	3.72	0.01	0.01	0.13
patient 18	0.14	0.31	0.97	0.14	5.46	28.58	4.25	0.01	0.01	0.28
patient 19	4.15	2.83	1.11	0.41	5.98	24.52	4.58	0.03	0.01	0.11
patient 20	1.07	1.11	1.27	0.23	7.51	20.50	4.25	0.01	0.01	0.17
patient 21	0.28	0.30	1.01	0.41	7.94	33.25	3.62	0.01	0.01	0.09
patient 22	0.78	0.44	0.93	1.30	4.81	30.02	3.97	0.01	0.01	0.16
patient 23	0.12	0.28	0.75	0.10	3.28	22.46	3.51	0.01	0.01	0.08
patient 24	0.08	0.64	1.17	0.92	4.14	17.55	3.39	0.01	0.01	0.09
patient 25	0.14	0.25	1.30	0.94	5.05	14.40	4.46	0.01	0.01	0.08
patient 26	0.61	0.54	0.66	2.41	3.68	15.70	4.42	0.01	0.01	0.17

© 2025 Rakow A et al. *JAMA Network Open*.

	Co	Cr	Mo	Ni	Ti	Al	V	Nb	Ta	Zr
patient 27	0.16	0.28	0.76	0.55	5.65	38.74	4.03	0.03	0.01	0.10
patient 28	0.21	0.55	1.03	0.46	6.25	32.44	4.56	0.01	0.01	0.11
patient 29	12.72	4.11	1.12	0.85	3.64	25.62	4.24	0.01	0.01	0.84
patient 30	1.25	1.35	1.04	0.39	4.96	28.84	5.24	0.32	0.01	56.88
patient 31	2.79	1.07	0.86	0.56	5.19	23.66	5.15	0.02	0.03	0.76
patient 32	0.17	0.29	1.23	0.62	3.90	23.87	4.60	0.07	0.01	0.67
patient 33	0.30	0.47	0.51	2.92	6.62	31.28	4.33	0.01	0.01	0.04
patient 34	0.19	0.34	1.01	1.19	8.63	20.49	3.93	0.08	0.30	0.08
patient 35	0.35	0.55	0.97	1.19	3.88	27.94	4.71	0.01	0.01	0.08
patient 36	0.23	0.35	1.03	1.33	3.94	20.70	4.15	0.01	0.01	0.05
patient 37	0.20	0.41	1.29	1.07	4.57	17.43	5.06	0.01	0.01	0.07
patient 38	0.12	1.29	1.09	0.69	5.07	31.43	3.99	0.14	0.01	9.46
patient 39	0.08	0.24	0.47	1.74	1.19	15.85	3.50	0.01	0.01	0.08
patient 40	0.25	0.22	0.94	0.34	1.98	14.47	4.91	0.02	0.01	0.13
patient 41	0.23	0.26	0.47	0.42	2.25	26.43	3.14	0.04	0.01	0.11
patient 42	0.14	0.54	1.21	1.44	8.42	23.65	7.03	0.09	0.01	0.08
patient 43	0.06	0.33	0.85	0.08	0.81	22.74	4.91	0.04	0.01	0.08
patient 44	0.22	1.60	0.70	1.67	2.22	25.79	2.55	0.04	0.01	0.26
patient 45	0.14	0.26	0.79	0.22	2.31	15.55	3.35	0.02	0.01	0.17
patient 46	0.17	1.63	2.89	4.94	3.66	35.87	25.49	0.04	0.01	0.10
patient 47	0.38	0.43	0.87	0.44	5.71	48.60	4.26	0.07	0.04	0.35
patient 48	0.25	0.27	1.47	0.79	2.92	32.49	4.04	0.05	0.03	0.18
patient 49	0.09	0.58	1.85	0.56	4.71	31.55	11.01	0.08	0.02	0.16
patient 50	0.13	0.39	0.66	0.78	4.80	19.77	3.61	0.13	0.09	0.11
patient 51	0.22	0.41	0.84	0.63	3.58	19.49	4.18	0.01	0.01	0.09
patient 52	0.08	0.41	1.24	3.47	8.45	24.16	5.50	0.01	0.01	0.09
patient 53	0.11	0.39	1.26	0.97	8.85	22.60	3.76	0.02	0.01	0.12
patient 54	0.13	0.47	1.05	0.71	4.12	32.27	4.05	0.01	0.02	0.20
patient 55	0.14	0.60	0.84	1.62	4.00	22.00	3.45	0.01	0.01	0.05

© 2025 Rakow A et al. *JAMA Network Open*.

	Co	Cr	Mo	Ni	Ti	Al	V	Nb	Ta	Zr
patient 56	0.56	0.47	0.63	0.65	4.66	30.51	0.37	0.01	0.01	0.10
patient 57	0.38	1.25	1.71	2.35	4.11	40.40	16.22	0.01	0.01	0.09
patient 58	0.23	0.61	0.93	0.66	3.30	30.49	6.40	0.01	0.01	0.14
patient 59	0.17	1.01	0.94	0.55	7.68	19.12	3.72	0.01	0.01	0.08
patient 60	0.35	0.28	1.06	0.88	3.03	23.64	3.81	0.01	0.02	0.14
patient 61	2.56	1.57	0.66	0.53	5.02	30.17	4.34	2.43	0.02	0.11
patient 62	0.14	0.49	0.76	1.60	4.55	22.81	4.77	0.04	0.02	0.13
patient 63	0.33	0.66	0.71	0.31	4.23	25.52	3.77	0.04	0.02	0.08
patient 64	0.18	0.45	0.72	0.64	2.17	25.07	4.05	0.03	0.01	0.08
patient 65	2.20	1.15	0.87	0.67	7.14	32.64	5.69	0.01	0.02	1.95
patient 66	1.56	0.61	0.56	0.57	7.42	23.74	4.57	0.75	0.01	0.30
patient 67	0.17	0.69	1.03	0.84	2.06	26.73	11.74	0.03	0.01	0.09
patient 68	2.18	1.32	0.95	1.09	7.71	31.17	5.71	0.01	0.01	2.27
patient 69	0.18	2.25	1.92	5.94	10.77	28.50	3.11	0.04	0.01	0.28
patient 70	7.79	3.99	1.49	1.31	8.33	43.36	8.30	0.01	0.01	2.89
patient 71	0.75	0.35	0.94	0.01	6.79	28.37	0.07	0.01	0.01	0.51
patient 72	0.18	0.65	1.20	4.61	8.36	40.72	7.20	0.01	0.01	0.01
patient 73	0.28	0.27	0.50	9.28	4.11	18.06	0.21	0.01	0.01	0.26
patient 74	0.07	0.32	0.65	0.30	4.47	17.48	3.86	0.01	0.01	0.14
patient 75	0.36	0.72	1.06	0.81	2.94	23.11	3.75	0.01	0.01	0.16
patient 76	0.18	0.28	1.88	5.27	4.41	15.91	4.09	0.01	0.01	0.11
patient 77	2.80	1.51	1.34	2.21	4.39	23.03	3.74	0.01	0.01	0.08
patient 78	0.74	0.57	0.83	0.50	4.80	18.82	3.42	0.01	0.01	0.24
patient 79	0.10	0.33	0.71	0.27	5.07	17.41	3.43	0.02	0.01	0.15
patient 80	29.03	8.15	1.88	0.46	4.76	19.79	3.99	0.01	0.01	0.17
patient 81	0.12	0.31	1.21	0.66	5.06	19.16	3.45	0.02	0.01	0.09
patient 82	0.10	0.42	0.97	0.97	3.90	27.43	4.19	0.02	0.01	0.13
patient 83	0.17	0.28	1.11	0.44	9.22	24.40	3.57	0.02	0.19	0.21
patient 84	2.12	0.93	1.01	0.40	88.21	23.47	4.28	0.05	0.01	0.19

© 2025 Rakow A et al. *JAMA Network Open*.

	Co	Cr	Mo	Ni	Ti	Al	V	Nb	Ta	Zr
patient 85	0.84	0.31	0.85	0.86	2.76	28.51	3.31	0.01	0.01	0.17
patient 86	0.45	1.18	0.58	0.29	3.67	28.14	3.73	0.01	0.01	0.10
patient 87	0.33	0.32	0.88	8.24	5.17	34.39	4.63	0.01	0.01	0.13
patient 88	0.45	0.67	1.27	0.23	5.49	24.78	4.61	0.01	0.01	0.11
patient 89	0.41	0.28	0.83	0.68	7.32	25.16	3.78	0.03	0.01	0.08
patient 90	0.18	0.46	0.85	0.38	5.39	18.63	3.93	0.01	0.01	0.09
patient 91	0.13	0.40	1.50	0.01	3.34	16.04	3.98	0.01	0.01	0.11
patient 92	0.13	0.23	0.94	0.86	9.89	17.01	3.51	0.03	0.03	0.12
patient 93	0.27	0.24	1.44	1.35	3.07	11.02	3.29	0.01	0.02	0.17
patient 94	0.12	0.38	1.06	1.94	2.93	18.37	3.70	0.01	0.01	0.11
patient 95	1.57	1.27	0.59	0.77	2.34	10.78	0.41	0.01	0.01	3.43
patient 96	6.45	3.35	0.51	2.07	3.32	15.10	0.33	0.04	0.01	1.14
patient 97	0.09	0.30	0.92	4.07	2.46	13.15	3.47	0.01	0.01	0.14
patient 98	0.79	0.64	1.28	5.81	5.35	16.09	4.55	0.01	0.01	0.23
patient 99	0.31	0.27	1.34	1.09	3.66	23.34	3.61	0.19	0.01	0.12
patient 100	0.41	0.27	0.76	0.98	13.89	33.02	3.69	0.01	0.01	0.11
patient 101	0.10	0.35	1.19	9.37	3.64	17.76	3.51	0.01	0.01	0.10
patient 102	0.20	0.51	1.11	1.72	2.42	17.91	4.35	0.01	0.01	0.09
**control group**										
patient 1	0.09	0.30	1.65	0.21	5.38	32.04	3.67	0.01	0.02	0.12
patient 2	0.04	0.40	1.37	0.35	3.83	29.69	3.96	0.01	0.01	0.17
patient 3	0.13	0.36	0.57	1.10	4.68	36.29	3.92	0.07	0.10	0.08
patient 4	0.34	0.37	0.89	0.18	2.42	18.32	4.43	0.12	0.01	0.11
patient 5	0.23	0.34	1.01	0.40	4.67	21.14	4.37	0.01	0.01	0.16
patient 6	0.15	0.44	1.61	0.01	5.84	38.71	6.19	0.01	0.01	0.01
patient 7	0.21	0.38	1.17	4.54	5.40	41.51	4.39	0.01	0.01	0.01
patient 8	0.14	0.39	0.80	0.36	5.67	22.96	4.56	0.01	0.01	0.14
patient 9	0.14	0.26	0.71	0.19	2.40	18.13	3.94	0.01	0.01	0.11
patient 10	0.27	0.39	1.11	0.75	3.35	17.98	5.24	0.01	0.01	0.13

© 2025 Rakow A et al. *JAMA Network Open*.

	Co	Cr	Mo	Ni	Ti	Al	V	Nb	Ta	Zr
patient 11	0.08	0.49	0.76	0.01	4.10	42.61	4.22	0.01	0.01	0.01
patient 12	0.08	0.37	0.98	0.48	2.84	25.00	3.82	0.01	0.01	0.10
patient 13	1.09	0.40	0.62	0.32	3.12	20.18	3.86	0.02	0.01	0.06
patient 14	0.14	0.59	1.33	1.14	5.09	37.15	7.81	0.10	0.13	0.33
patient 15	0.23	0.40	1.15	1.16	3.73	29.09	4.88	0.01	0.02	0.11
patient 16	0.15	2.44	1.10	0.34	4.65	33.96	6.09	0.09	0.01	0.18
patient 17	0.16	1.04	1.17	2.54	2.90	35.38	4.99	0.01	0.02	0.14
patient 18	0.21	0.24	0.88	1.14	4.35	32.39	4.17	0.01	0.01	0.04
patient 19	0.10	0.43	2.20	0.50	4.76	15.91	3.15	0.01	0.01	0.12
patient 20	0.45	0.23	1.20	0.41	4.99	17.26	3.56	0.01	0.01	0.07
patient 21	0.11	0.30	1.11	0.49	2.78	25.33	3.68	0.02	0.01	0.07
patient 22	0.14	0.35	0.85	0.53	3.66	19.67	4.37	0.01	0.01	0.10
patient 23	0.13	0.17	1.04	0.65	4.69	33.56	3.78	0.11	0.02	0.22
patient 24	0.04	0.21	1.10	0.27	4.72	25.54	4.52	0.01	0.01	0.08
patient 25	0.15	0.46	1.60	0.01	7.11	45.67	7.21	0.01	0.01	0.01
patient 26	0.07	0.23	1.03	0.46	2.95	20.81	4.40	0.03	0.03	0.06
patient 27	0.15	0.28	0.65	0.67	1.21	19.60	3.96	0.01	0.01	0.08
patient 28	0.12	0.32	0.81	0.58	1.89	26.33	5.23	0.01	0.01	0.07
patient 29	0.22	0.38	0.89	1.50	4.04	26.87	3.87	0.04	0.02	0.10
patient 30	0.09	0.23	0.45	0.14	2.69	17.68	3.50	0.01	0.01	0.04
patient 31	0.08	0.40	1.62	0.49	3.14	31.28	3.77	0.01	0.01	0.12
patient 32	0.16	0.32	1.06	0.29	4.38	21.65	3.87	0.01	0.01	0.10
patient 33	0.18	0.56	1.09	2.28	3.88	17.91	4.73	0.01	0.01	0.15
patient 34	0.07	0.32	1.38	1.22	3.80	22.28	4.73	0.01	0.02	0.11
patient 35	0.18	0.35	1.16	1.11	4.65	19.51	5.03	0.01	0.01	0.10
patient 36	0.21	0.35	1.59	0.69	5.77	21.18	4.77	0.02	0.02	0.17
patient 37	0.12	0.26	1.03	0.50	1.62	21.03	3.60	0.04	0.03	0.06
patient 38	0.12	0.31	1.50	0.55	5.79	18.65	4.53	0.01	0.01	0.05
patient 39	0.09	0.43	0.77	0.95	3.19	19.92	5.33	0.01	0.02	0.12

© 2025 Rakow A et al. *JAMA Network Open*.

	Co	Cr	Mo	Ni	Ti	Al	V	Nb	Ta	Zr
patient 40	0.09	0.46	1.26	1.07	3.00	16.57	4.71	0.01	0.02	0.14
patient 41	0.12	0.35	0.88	1.13	3.63	22.27	4.30	0.01	0.01	0.10
patient 42	0.19	0.37	0.84	6.65	4.41	27.60	4.98	0.01	0.02	0.07
patient 43	0.19	0.24	1.05	0.83	3.12	32.26	4.75	0.06	0.01	0.13
patient 44	0.07	0.38	0.56	0.18	3.14	29.30	3.18	0.05	0.01	0.14
patient 45	0.07	0.25	0.91	0.26	3.99	22.55	3.42	0.01	0.01	0.04
patient 46	0.12	0.33	0.71	0.20	4.62	30.82	3.91	0.01	0.01	0.36
patient 47	0.14	0.29	1.15	1.46	2.61	17.52	3.51	0.02	0.01	0.09
patient 48	0.16	0.36	1.68	0.65	6.60	30.20	3.98	0.01	0.01	0.12
patient 49	0.09	0.27	1.03	0.25	2.89	16.15	4.02	0.02	0.01	0.05
patient 50	1.21	0.38	0.94	1.13	6.15	34.85	4.41	0.05	0.16	0.15
patient 51	0.24	0.44	1.07	0.55	4.59	27.91	4.71	0.01	0.01	0.09
patient 52	0.18	0.29	1.03	0.50	3.40	30.50	7.05	0.02	0.01	0.19
patient 53	0.08	0.41	1.29	1.02	4.99	18.75	4.08	0.01	0.01	0.11
patient 54	0.15	0.37	1.22	1.31	2.94	15.66	4.01	0.01	0.01	0.10
patient 55	0.12	0.58	1.11	1.44	4.53	28.45	6.19	0.01	0.01	0.07
patient 56	0.08	0.35	0.86	1.33	2.76	13.84	4.44	0.01	0.05	0.13
patient 57	0.07	0.24	1.08	0.49	4.22	18.77	4.04	0.01	0.03	0.09
patient 58	0.08	0.27	0.85	0.45	5.19	19.42	3.61	0.02	0.01	0.05
patient 59	0.17	0.33	1.32	0.90	3.96	12.20	3.57	0.01	0.01	0.11
patient 60	0.49	0.79	1.92	2.05	3.45	23.94	8.31	0.01	0.01	0.09
patient 61	0.09	0.72	1.16	0.01	3.05	34.92	4.09	0.01	0.01	0.01
patient 62	0.16	0.29	1.08	1.30	2.56	13.62	3.33	0.01	0.01	0.10
patient 63	0.09	0.34	1.02	11.47	3.83	27.88	3.71	0.02	0.02	0.04
patient 64	0.10	0.22	1.62	0.34	3.41	25.01	4.16	0.01	0.01	0.04
patient 65	0.09	0.27	0.55	0.13	2.27	15.84	4.09	0.01	0.01	0.05
patient 66	0.15	0.26	2.70	0.31	3.02	34.83	3.38	0.01	0.01	0.07
patient 67	0.12	0.37	1.40	0.53	3.46	28.69	7.64	0.02	0.01	0.15
patient 68	0.10	0.25	1.44	0.40	3.14	20.20	3.76	0.01	0.01	0.06

© 2025 Rakow A et al. *JAMA Network Open*.

	Co	Cr	Mo	Ni	Ti	Al	V	Nb	Ta	Zr
patient 69	0.16	0.35	0.91	1.42	2.51	10.66	3.99	0.01	0.03	0.09
patient 70	0.11	0.44	1.12	0.50	2.48	24.93	6.94	0.07	0.06	0.19
patient 71	0.13	0.36	0.58	1.47	2.27	28.08	4.70	0.02	0.01	0.05
patient 72	0.48	0.48	0.62	1.48	6.13	23.44	4.20	0.13	0.01	0.07
patient 73	0.28	0.54	0.94	3.92	4.71	18.14	4.06	0.07	0.03	0.13
patient 74	0.15	0.33	1.12	0.52	5.32	33.74	4.60	0.01	0.02	0.06
patient 75	0.06	0.37	1.41	0.43	5.92	27.23	3.99	0.07	0.13	0.33
patient 76	0.08	0.21	1.21	0.25	3.35	12.55	3.29	0.01	0.01	0.05
patient 77	1.08	0.29	0.25	0.11	3.10	13.40	3.29	0.02	0.01	0.09
patient 78	0.18	0.38	1.23	0.30	4.03	21.21	3.97	0.01	0.01	0.07
patient 79	0.12	0.37	0.98	0.66	3.60	25.46	4.30	0.01	0.01	0.12
patient 80	0.22	0.49	1.31	0.79	4.34	24.52	3.45	0.01	0.01	0.13
patient 81	0.10	0.64	0.90	0.46	3.90	35.94	4.99	0.01	0.01	0.08
patient 82	0.05	0.26	0.56	0.26	0.19	13.15	3.42	0.01	0.01	0.09
patient 83	0.09	0.32	0.75	1.07	2.50	13.73	3.75	0.01	0.02	0.11
patient 84	0.13	0.30	1.03	0.88	3.29	18.88	3.67	0.01	0.01	0.06
patient 85	0.09	0.55	1.18	1.11	4.13	17.70	4.94	0.01	0.02	0.08
patient 86	0.11	0.37	0.89	0.55	5.44	33.04	4.46	0.02	0.01	0.05
patient 87	0.27	0.57	1.57	1.10	3.83	34.12	7.07	0.01	0.01	0.09
patient 88	0.12	0.30	1.47	0.60	7.86	21.88	4.21	0.01	0.01	0.09
patient 89	0.12	0.33	0.48	0.67	1.80	18.73	3.49	0.01	0.01	0.04
patient 90	0.60	0.30	0.67	0.13	3.38	14.68	2.96	0.01	0.01	0.08
patient 91	0.20	0.36	1.32	0.01	5.14	40.39	4.24	0.01	0.01	0.01
patient 92	0.16	0.53	0.91	0.83	4.58	27.16	4.33	0.01	0.01	0.12
patient 93	0.15	0.32	1.30	0.43	3.19	25.98	3.73	0.01	0.01	0.09
patient 94	0.07	0.35	1.10	4.96	4.73	32.15	7.03	0.03	0.01	0.08
patient 95	0.07	0.43	0.94	0.01	4.43	38.69	3.50	0.01	0.01	0.01
patient 96	0.12	0.42	0.61	0.29	4.07	33.38	4.51	0.01	0.01	0.28
patient 97	0.17	0.36	0.94	9.75	2.39	18.44	4.04	0.01	0.01	0.13

© 2025 Rakow A et al. *JAMA Network Open*.

	Co	Cr	Mo	Ni	Ti	Al	V	Nb	Ta	Zr
patient 98	0.08	0.43	1.01	0.40	7.27	25.97	4.37	0.01	0.01	0.06
patient 99	0.09	0.32	1.20	0.40	3.83	32.56	5.21	0.05	0.03	0.16
patient 100	0.24	0.35	1.41	0.44	3.34	24.97	4.61	0.01	0.01	0.06
patient 101	0.09	0.26	0.97	0.53	3.18	22.71	5.86	0.02	0.01	0.11
patient 102	0.21	0.37	0.91	0.21	4.15	18.77	4.97	0.01	0.01	0.13

© 2025 Rakow A et al. *JAMA Network Open*.

eTable 6. Metal levels [µg/L] Quantified by Inductively Coupled Plasma Mass Spectrometry in Cerebrospinal Fluid of Patients With at Least 1 Arthroplasty Implant in Situ and of Age- and Sex-Matched Arthroplasty Implant-Naive Patients

	Co	Cr	Mo	Ni	Ti	Al	V	Nb	Ta	Zr
**implant group**										
patient 1	0.03	0.60	0.29	0.49	0.91	10.61	0.19	0.01	0.01	0.02
patient 2	0.01	0.08	0.10	0.12	0.55	11.30	0.19	0.01	0.01	0.02
patient 3	0.02	0.03	0.23	0.01	0.64	7.69	0.15	0.01	0.01	0.02
patient 4	0.02	0.14	0.13	0.23	0.65	17.73	0.14	0.01	0.01	0.10
patient 5	0.02	0.36	0.11	0.33	0.50	10.85	0.24	0.02	0.01	0.05
patient 6	0.03	0.11	0.17	0.29	0.68	9.98	0.05	0.01	0.01	0.01
patient 7	0.02	0.05	0.05	0.07	0.10	9.29	0.09	0.01	0.01	0.04
patient 8	0.03	0.12	0.05	0.10	0.21	8.97	0.12	0.01	0.01	0.05
patient 9	0.03	0.33	0.23	0.79	0.47	17.54	0.53	0.01	0.01	0.03
patient 10	0.30	0.27	0.23	0.26	0.43	15.75	0.52	0.02	0.01	0.04
patient 11	0.02	0.22	0.18	0.45	0.54	13.49	0.11	0.01	0.01	0.02
patient 12	0.06	1.06	0.15	1.13	0.12	10.57	0.07	0.01	0.01	0.01
patient 13	0.05	0.18	0.21	0.39	0.38	12.02	0.08	0.01	0.01	0.01
patient 14	0.01	0.23	0.21	0.43	0.17	11.96	0.05	0.01	0.01	0.01
patient 15	0.05	0.40	0.10	0.94	0.68	14.46	0.06	0.01	0.01	0.01
patient 16	0.06	0.61	0.12	1.00	0.88	20.90	0.13	0.01	0.01	0.06
patient 17	0.03	0.24	0.10	0.66	1.07	8.64	0.13	0.01	0.01	0.06
patient 18	0.03	0.34	0.21	0.80	0.88	9.35	0.13	0.01	0.01	0.05
patient 19	0.20	0.17	0.26	0.40	1.10	7.53	0.10	0.01	0.01	0.04
patient 20	0.04	0.23	0.13	0.41	0.88	7.58	0.10	0.01	0.01	0.04
patient 21	0.04	0.45	0.21	0.84	0.87	6.93	0.11	0.01	0.01	0.03
patient 22	0.06	0.18	0.15	0.42	0.94	8.05	0.11	0.01	0.01	0.06
patient 23	0.04	1.86	0.13	0.96	0.88	9.51	0.08	0.01	0.01	0.03
patient 24	0.04	0.19	0.22	0.99	0.57	5.41	0.05	0.01	0.01	0.05
patient 25	0.04	0.59	0.17	1.40	0.56	7.64	0.07	0.01	0.01	0.02
patient 26	0.03	0.31	0.05	0.89	0.73	7.43	0.20	0.01	0.01	0.05

© 2025 Rakow A et al. *JAMA Network Open*.

	Co	Cr	Mo	Ni	Ti	Al	V	Nb	Ta	Zr
patient 27	0.05	0.78	0.12	2.04	0.85	19.98	0.24	0.05	0.01	0.04
patient 28	0.02	0.07	0.17	1.00	0.90	9.46	0.24	0.01	0.01	0.03
patient 29	0.28	0.25	0.20	2.17	0.73	8.52	0.28	0.01	0.01	0.04
patient 30	0.04	0.28	0.12	0.60	0.65	18.09	0.27	0.03	0.01	0.44
patient 31	0.14	0.40	0.15	1.95	1.04	14.65	0.32	0.03	0.01	0.06
patient 32	0.07	1.04	0.30	2.82	0.76	15.87	0.30	0.16	0.01	0.04
patient 33	0.02	0.37	0.06	0.46	0.60	12.88	0.21	0.07	0.01	0.01
patient 34	0.05	0.36	0.14	1.03	1.06	31.11	0.12	0.02	0.01	0.02
patient 35	0.04	0.87	0.07	0.96	0.53	19.60	0.12	0.05	0.01	0.02
patient 36	0.03	0.15	0.12	0.79	0.77	9.88	0.06	0.01	0.01	0.03
patient 37	0.04	0.60	0.18	1.41	0.73	11.51	0.06	0.01	0.01	0.02
patient 38	0.02	0.10	0.24	0.56	0.66	9.12	0.05	0.01	0.01	0.09
patient 39	0.07	0.47	0.07	1.59	0.41	10.11	0.12	0.02	0.01	0.02
patient 40	0.04	1.17	0.20	0.91	0.31	11.51	0.21	0.01	0.01	0.04
patient 41	0.04	0.28	0.27	0.99	2.90	54.96	0.89	0.02	0.01	0.05
patient 42	0.02	0.82	0.08	0.41	0.34	9.23	0.19	0.01	0.01	0.33
patient 43	0.01	0.11	0.05	0.34	0.18	7.89	0.16	0.01	0.01	0.08
patient 44	0.02	0.14	0.47	0.19	0.27	6.43	0.09	0.01	0.01	0.05
patient 45	0.02	0.08	0.19	0.28	0.87	13.55	0.11	0.01	0.01	0.09
patient 46	0.02	0.07	0.03	0.21	0.32	7.96	0.07	0.01	0.01	0.04
patient 47	0.03	0.66	0.14	0.92	0.50	8.02	0.35	0.03	0.01	0.05
patient 48	0.02	0.06	0.24	0.14	0.28	7.59	0.31	0.01	0.01	0.08
patient 49	0.02	0.06	0.21	0.19	0.29	10.69	0.53	0.01	0.01	0.04
patient 50	0.04	0.42	0.11	0.56	0.54	10.18	0.61	0.08	0.01	0.02
patient 51	0.03	0.60	0.12	1.06	0.50	9.53	0.13	0.01	0.01	0.03
patient 52	0.03	0.91	0.19	1.15	0.49	9.77	0.15	0.01	0.01	0.01
patient 53	0.03	0.97	0.16	0.97	0.55	11.74	0.13	0.03	0.01	0.01
patient 54	0.04	0.81	0.13	0.75	0.58	11.61	0.10	0.01	0.01	0.05
patient 55	0.05	0.75	0.11	1.31	0.52	17.57	0.16	0.01	0.01	0.01

© 2025 Rakow A et al. *JAMA Network Open*.

	Co	Cr	Mo	Ni	Ti	Al	V	Nb	Ta	Zr
patient 56	0.02	0.08	0.13	0.29	0.45	11.57	0.13	0.01	0.01	0.02
patient 57	0.02	0.06	0.21	0.16	0.45	11.82	0.09	0.01	0.01	0.02
patient 58	0.01	0.02	0.07	0.10	0.59	9.68	0.12	0.01	0.01	0.01
patient 59	0.02	0.37	0.15	0.47	0.65	11.42	0.17	0.02	0.01	0.02
patient 60	0.02	0.15	0.15	0.23	0.32	13.36	0.08	0.02	0.01	0.02
patient 61	0.26	0.40	0.19	0.68	0.67	14.53	0.12	0.07	0.01	0.01
patient 62	0.03	0.28	0.11	0.87	0.56	13.53	0.07	0.06	0.01	0.03
patient 63	0.03	0.41	0.08	0.47	0.55	9.63	0.07	0.03	0.01	0.02
patient 64	0.02	0.22	0.17	0.24	0.36	13.87	0.07	0.02	0.01	0.02
patient 65	0.07	0.33	0.15	0.44	0.46	10.27	0.05	0.01	0.01	0.03
patient 66	0.10	0.80	0.16	1.15	0.24	17.07	0.08	0.05	0.01	0.03
patient 67	0.05	1.05	0.09	1.09	0.49	36.63	0.18	0.26	0.01	0.03
patient 68	0.07	0.07	0.16	0.21	0.36	10.72	0.06	0.02	0.01	0.03
patient 69	0.03	0.43	0.54	0.70	0.82	14.05	0.13	0.01	0.01	0.05
patient 70	0.17	0.18	0.14	0.11	0.83	13.81	0.06	0.01	0.01	0.05
patient 71	0.03	0.22	0.19	0.34	0.51	13.81	0.06	0.01	0.01	0.02
patient 72	0.02	0.10	0.08	0.63	0.75	18.18	0.06	0.01	0.01	0.02
patient 73	0.06	1.26	0.16	1.40	1.02	14.38	0.48	0.02	0.01	0.09
patient 74	0.03	0.26	0.11	0.48	1.41	9.27	0.30	0.01	0.01	0.04
patient 75	0.07	0.28	0.16	2.51	0.78	6.89	0.32	0.02	0.01	0.07
patient 76	0.02	0.22	0.35	0.51	0.99	14.20	0.27	0.01	0.01	0.04
patient 77	0.21	0.07	0.33	0.81	0.89	10.23	0.25	0.01	0.01	0.04
patient 78	0.05	0.84	0.13	0.77	0.82	11.00	0.33	0.01	0.01	0.05
patient 79	0.02	0.07	0.08	0.73	0.84	9.92	0.27	0.01	0.01	0.04
patient 80	0.64	0.42	0.29	0.93	1.09	9.46	0.33	0.01	0.01	0.04
patient 81	0.03	0.49	0.24	0.73	0.85	9.74	0.32	0.02	0.01	0.04
patient 82	0.02	0.18	0.18	0.56	1.06	7.42	0.28	0.01	0.01	0.04
patient 83	0.02	0.17	0.14	0.99	0.87	7.64	0.31	0.01	0.01	0.05
patient 84	0.07	0.48	0.20	0.87	1.40	10.84	0.42	0.16	0.01	0.05

© 2025 Rakow A et al. *JAMA Network Open*.

	Co	Cr	Mo	Ni	Ti	Al	V	Nb	Ta	Zr
patient 85	0.03	0.49	0.12	1.10	0.84	9.60	0.28	0.02	0.01	0.04
patient 86	0.04	0.13	0.08	0.50	1.00	8.99	0.25	0.01	0.01	0.02
patient 87	0.03	0.88	0.15	0.73	0.94	9.10	0.17	0.02	0.01	0.05
patient 88	0.03	0.07	0.15	0.89	0.88	8.34	0.21	0.01	0.01	0.04
patient 89	0.04	0.22	0.10	0.57	1.22	10.17	0.24	0.01	0.01	0.04
patient 90	0.02	0.14	0.09	0.53	1.61	9.90	0.23	0.01	0.01	0.17
patient 91	0.02	0.35	0.16	0.54	0.96	7.20	0.25	0.03	0.01	0.12
patient 92	0.03	0.11	0.17	0.40	0.91	5.06	0.29	0.01	0.01	0.08
patient 93	0.03	0.03	0.65	0.59	0.78	4.94	0.33	0.01	0.01	0.06
patient 94	0.02	0.14	0.15	0.64	0.79	6.15	0.26	0.01	0.01	0.06
patient 95	0.09	0.16	0.12	1.39	0.44	6.49	0.16	0.01	0.01	0.08
patient 96	0.19	0.19	0.14	1.46	0.66	6.35	0.14	0.01	0.01	0.05
patient 97	0.02	0.32	0.13	0.97	0.66	5.59	0.17	0.01	0.01	0.05
patient 98	0.04	0.56	0.34	0.99	0.60	5.27	0.34	0.02	0.01	0.04
patient 99	0.05	2.05	0.28	1.65	1.30	6.25	0.28	0.01	0.01	0.05
patient 100	0.02	0.10	0.17	0.68	0.74	4.40	0.22	0.01	0.01	0.04
patient 101	0.07	0.59	0.17	1.17	0.40	8.29	0.11	0.01	0.01	0.05
patient 102	0.05	0.32	0.14	0.97	0.61	9.83	0.65	0.01	0.01	0.05
**control group**										
patient 1	0.02	0.15	0.18	0.37	0.92	7.75	0.11	0.01	0.01	0.06
patient 2	0.02	0.14	0.19	0.40	0.90	6.18	0.11	0.01	0.01	0.03
patient 3	0.02	0.52	0.07	0.47	0.26	13.02	0.07	0.07	0.01	0.04
patient 4	0.02	0.23	0.03	0.60	0.33	16.65	0.07	0.03	0.01	0.05
patient 5	0.05	0.37	0.10	0.78	0.57	11.24	0.08	0.01	0.01	0.01
patient 6	0.02	0.04	0.11	0.43	0.96	17.42	0.05	0.01	0.01	0.01
patient 7	0.02	0.12	0.06	1.23	0.52	18.21	0.04	0.01	0.01	0.01
patient 8	0.02	0.45	0.18	0.65	1.30	9.44	0.35	0.01	0.01	0.06
patient 9	0.02	0.22	0.17	0.35	1.00	10.29	0.40	0.03	0.01	0.05
patient 10	0.03	0.12	0.18	0.88	1.10	9.56	0.31	0.01	0.01	0.06

© 2025 Rakow A et al. *JAMA Network Open*.

	Co	Cr	Mo	Ni	Ti	Al	V	Nb	Ta	Zr
patient 11	0.03	0.44	0.06	0.99	0.47	14.65	0.07	0.01	0.01	0.01
patient 12	0.03	0.41	0.11	0.81	0.44	12.90	0.11	0.02	0.01	0.02
patient 13	0.19	0.88	0.16	0.59	0.20	12.23	0.09	0.03	0.01	0.02
patient 14	0.04	0.73	0.09	1.06	0.25	11.86	0.46	0.03	0.01	0.03
patient 15	0.03	0.19	0.21	0.55	1.36	15.99	0.28	0.01	0.01	0.04
patient 16	0.02	0.29	0.13	0.42	0.58	11.48	0.17	0.02	0.01	0.03
patient 17	0.02	0.99	0.15	0.77	0.51	9.78	0.11	0.01	0.01	0.05
patient 18	0.02	0.33	0.09	0.62	0.92	16.52	0.23	0.07	0.01	0.02
patient 19	0.02	0.05	0.80	0.23	0.77	9.31	0.05	0.01	0.01	0.03
patient 20	0.04	0.14	0.21	0.23	0.64	9.47	0.05	0.01	0.01	0.01
patient 21	0.04	1.11	0.21	1.26	0.20	12.08	0.09	0.07	0.01	0.02
patient 22	0.02	0.05	0.11	0.43	0.85	8.23	0.23	0.01	0.01	0.04
patient 23	0.02	0.23	0.13	0.47	0.40	9.94	0.17	0.04	0.01	0.05
patient 24	0.02	0.31	0.15	0.94	1.45	5.27	0.11	0.01	0.01	0.04
patient 25	0.05	0.98	0.08	1.28	0.55	17.54	0.05	0.01	0.01	0.01
patient 26	0.04	0.27	0.23	0.40	0.27	11.44	0.06	0.01	0.01	0.01
patient 27	0.01	0.04	0.01	0.20	0.18	8.50	0.10	0.01	0.01	0.07
patient 28	0.03	0.31	0.08	0.61	0.25	13.40	0.05	0.01	0.01	0.01
patient 29	0.02	0.10	0.15	1.10	0.72	32.03	0.27	0.01	0.01	0.28
patient 30	0.01	0.08	0.07	0.15	0.21	10.66	0.03	0.01	0.01	0.01
patient 31	0.02	0.28	0.36	0.56	0.76	9.04	0.35	0.03	0.01	0.04
patient 32	0.04	0.14	0.20	0.66	1.52	6.63	0.07	0.01	0.01	0.04
patient 33	0.01	0.06	0.11	0.52	0.72	4.97	0.23	0.01	0.01	0.07
patient 34	0.02	0.63	0.24	0.87	0.42	6.36	0.25	0.01	0.01	0.05
patient 35	0.02	0.28	0.15	0.68	0.70	10.32	0.07	0.01	0.01	0.02
patient 36	0.03	0.07	0.31	0.45	0.39	9.57	0.06	0.01	0.01	0.08
patient 37	0.02	0.29	0.26	0.25	0.21	15.11	0.09	0.11	0.01	0.02
patient 38	0.01	0.04	0.10	0.06	0.78	10.64	0.06	0.01	0.01	0.03
patient 39	0.01	0.23	0.06	0.30	0.49	10.27	0.10	0.01	0.01	0.03

© 2025 Rakow A et al. *JAMA Network Open*.

	Co	Cr	Mo	Ni	Ti	Al	V	Nb	Ta	Zr
patient 40	0.01	0.21	0.15	0.56	1.10	6.08	0.32	0.01	0.01	0.06
patient 41	0.03	0.44	0.39	0.30	0.32	12.60	0.12	0.17	0.01	0.01
patient 42	0.02	0.07	0.12	0.20	0.68	11.32	0.04	0.02	0.01	0.02
patient 43	0.17	0.91	0.14	2.64	0.93	13.07	0.19	0.01	0.01	0.05
patient 44	0.04	0.76	0.08	1.39	0.54	8.41	0.33	0.02	0.01	0.11
patient 45	0.05	0.98	0.19	0.87	0.30	11.13	0.03	0.01	0.01	0.02
patient 46	0.03	0.51	0.06	0.55	0.93	10.29	0.14	0.01	0.01	0.05
patient 47	0.04	0.23	0.13	0.72	0.13	11.81	0.15	0.02	0.01	0.04
patient 48	0.02	0.12	0.26	0.28	0.50	9.72	0.10	0.01	0.01	0.04
patient 49	0.01	0.09	0.01	0.74	0.36	9.42	0.08	0.02	0.01	0.04
patient 50	0.05	0.19	0.08	0.61	0.59	10.00	0.06	0.01	0.01	0.01
patient 51	0.05	0.80	0.20	1.35	1.62	8.39	0.43	0.01	0.01	0.05
patient 52	0.01	0.05	0.16	0.24	0.56	11.11	0.20	0.01	0.01	0.07
patient 53	0.02	0.06	0.17	0.69	0.68	4.14	0.05	0.01	0.01	0.05
patient 54	0.02	0.04	0.20	0.50	0.58	6.23	0.24	0.01	0.01	0.04
patient 55	0.01	0.05	0.08	0.19	0.50	10.01	0.12	0.01	0.01	0.01
patient 56	0.02	0.24	0.13	0.93	0.80	8.12	0.14	0.01	0.01	0.04
patient 57	0.03	0.52	0.17	0.47	0.52	10.93	0.30	0.06	0.01	0.08
patient 58	0.05	0.46	0.10	0.67	0.41	10.26	0.36	0.03	0.01	0.11
patient 59	0.03	0.31	0.35	0.94	0.68	5.82	0.21	0.01	0.01	0.06
patient 60	0.04	0.84	0.35	0.90	1.09	35.19	0.40	0.10	0.01	0.03
patient 61	0.03	0.11	0.46	0.77	0.26	14.17	0.09	0.01	0.01	0.02
patient 62	0.05	0.64	0.24	1.56	1.26	12.50	0.58	0.03	0.01	0.05
patient 63	0.03	0.36	0.18	0.47	0.46	7.96	0.36	0.02	0.01	0.06
patient 64	0.02	0.08	0.15	0.11	0.18	12.96	0.04	0.01	0.01	0.01
patient 65	0.01	0.08	0.05	0.12	0.30	10.29	0.05	0.01	0.01	0.01
patient 66	0.03	0.07	0.69	0.29	0.39	13.13	0.06	0.01	0.01	0.01
patient 67	0.03	0.46	0.46	1.15	0.85	13.19	0.33	0.01	0.01	0.04
patient 68	0.02	0.17	0.16	0.59	0.63	9.53	0.05	0.01	0.01	0.02

© 2025 Rakow A et al. *JAMA Network Open*.

	Co	Cr	Mo	Ni	Ti	Al	V	Nb	Ta	Zr
patient 69	0.04	0.38	0.21	0.81	0.57	7.49	0.40	0.04	0.01	0.14
patient 70	0.05	0.62	0.40	1.73	0.56	10.83	0.46	0.01	0.01	0.05
patient 71	0.02	0.04	0.10	0.23	0.18	11.53	0.06	0.01	0.01	0.02
patient 72	0.02	0.39	0.05	0.57	1.05	15.19	0.19	0.03	0.01	0.01
patient 73	0.03	0.14	0.15	0.36	0.42	9.60	0.43	0.01	0.01	0.06
patient 74	0.02	0.25	0.16	0.53	0.38	10.70	0.06	0.01	0.01	0.01
patient 75	0.03	0.11	0.24	0.49	0.43	14.14	0.58	0.02	0.01	0.03
patient 76	0.05	0.23	0.10	0.93	0.58	9.47	0.05	0.01	0.01	0.01
patient 77	0.04	0.53	0.12	1.14	0.01	14.37	0.14	0.02	0.01	0.04
patient 78	0.04	0.10	0.19	0.34	0.97	5.85	0.10	0.01	0.01	0.02
patient 79	0.01	0.03	0.14	0.28	1.40	8.85	0.10	0.01	0.01	0.05
patient 80	0.02	0.38	0.21	1.00	1.08	8.50	0.21	0.01	0.01	0.04
patient 81	0.02	0.25	0.13	0.58	0.51	22.42	0.12	0.01	0.01	0.02
patient 82	0.02	0.31	0.09	7.83	0.50	13.76	0.13	0.03	0.01	0.05
patient 83	0.03	2.62	0.18	1.28	0.49	5.70	0.27	0.01	0.01	0.06
patient 84	0.02	0.09	0.19	0.47	0.65	3.48	0.06	0.01	0.01	0.02
patient 85	0.02	0.48	0.16	0.63	0.86	6.59	0.30	0.01	0.01	0.04
patient 86	0.02	0.28	0.13	0.62	0.61	11.40	0.17	0.01	0.01	0.01
patient 87	0.03	0.24	0.23	0.48	1.35	8.39	0.27	0.01	0.01	0.04
patient 88	0.02	0.14	0.18	1.36	0.97	7.42	0.10	0.01	0.01	0.03
patient 89	0.02	0.18	0.04	0.45	0.23	11.60	0.05	0.01	0.01	0.02
patient 90	0.03	0.29	0.07	0.54	0.17	7.96	0.13	0.03	0.01	0.04
patient 91	0.02	0.12	0.19	0.32	0.49	13.65	0.09	0.01	0.01	0.03
patient 92	0.03	1.00	0.12	0.80	0.48	11.35	0.28	0.01	0.01	0.03
patient 93	0.03	0.44	0.16	0.53	0.57	10.59	0.13	0.01	0.01	0.02
patient 94	0.01	0.22	0.19	0.81	0.70	13.69	0.25	0.03	0.01	0.06
patient 95	0.02	0.14	0.15	0.40	0.52	17.96	0.06	0.01	0.01	0.02
patient 96	0.04	0.77	0.12	0.95	0.30	9.73	0.06	0.01	0.01	0.01
patient 97	0.02	0.20	0.20	0.62	1.08	11.58	0.26	0.01	0.01	0.04

© 2025 Rakow A et al. *JAMA Network Open*.

	Co	Cr	Mo	Ni	Ti	Al	V	Nb	Ta	Zr
patient 98	0.02	0.20	0.14	0.53	1.14	7.04	0.10	0.01	0.01	0.04
patient 99	0.01	0.02	0.13	0.18	0.56	8.20	0.38	0.01	0.01	0.04
patient 100	0.03	0.19	0.14	0.64	0.80	9.41	0.13	0.02	0.01	0.01
patient 101	0.02	0.22	0.12	0.50	0.70	12.40	0.22	0.02	0.01	0.05
patient 102	0.04	0.45	0.19	1.30	1.25	38.02	0.31	0.02	0.01	0.04

© 2025 Rakow A et al. *JAMA Network Open*.

eTable 7. Median and Maximum Metal Levels [µg/L] of 102 Patients With Arthroplasty Implant, 68 Patients With at Least 1 CoCrMo Component, 28 Patients With Arthroplasty Implant Without a CoCrMo Component, and Matched Controls

analyte	implant		control		p-value	CoCrMo		control		p-value	CoCrMo-free		control		p-value
	median	max	median	max		median	max	median	max		median	max	median	max	
**whole blood**															
Co	0.27	24.1	0.16	0.99	< .001	0.35	24.1	0.16	0.99	< .001	0.19	0.73	0.16	0.74	.90
Cr	0.47	4.76	0.42	1.52	< .001	0.48	4.76	0.42	1.52	< .001	0.43	1.81	0.40	0.52	.17
Mo	0.50	1.60	0.50	2.40	.19	0.50	1.20	0.60	2.40	.02	0.50	1.20	0.50	0.80	.37
Ni	1.00	17.3	0.90	63.3	.19	1.00	17.3	0.90	63.3	.64	1.15	5.00	0.80	1.80	.02
Ti	8.05	37.2	7.15	20.7	.04	7.60	37.2	7.15	20.7	.34	8.30	12.8	7.20	14.8	.12
Al	6.25	24.1	6.53	65.4	.92	6.38	24.1	6.53	65.4	.70	5.94	10.7	6.32	10.2	.60
V	0.06	0.81	0.06	0.19	.98	0.06	0.81	0.06	0.19	.96	0.04	0.30	0.06	0.09	.98
Nb	0.02	1.14	0.01	0.11	< .001	0.01	1.14	0.01	0.11	.01	0.03	0.09	0.01	0.10	.008
Ta	0.01	0.59	0.01	0.07	.04	0.01	0.20	0.01	0.07	.09	0.01	0.16	0.01	0.03	.59
Zr	0.05	39.9	0.03	1.95	< .001	0.06	39.9	0.03	0.80	.001	0.04	0.65	0.02	1.95	.38
**serum**															
Co	0.21	29.0	0.13	1.21	< .001	0.29	29.0	0.13	1.21	< .001	0.17	0.41	0.12	0.48	.15
Cr	0.42	8.15	0.36	2.44	< .001	0.47	8.15	0.36	1.04	< .001	0.34	1.60	0.33	2.44	.29
Mo	0.99	2.89	1.06	2.70	.18	0.96	2.89	1.09	2.70	.03	1.05	1.88	1.03	1.50	.50
Ni	0.68	9.37	0.53	11.5	.05	0.69	9.37	0.53	11.5	.11	0.73	5.27	0.53	6.65	.18
Ti	4.74	88.2	3.83	7.86	< .001	4.18	88.2	3.86	7.86	.17	5.08	13.9	3.62	6.13	< .001
Al	24.3	48.6	24.2	45.7	.82	23.8	48.6	24.7	41.5	.92	24.3	40.7	22.9	42.6	.39
V	4.01	25.5	4.21	8.31	.04	3.99	25.5	4.23	8.31	.06	3.96	11.0	4.21	7.05	.31
Nb	0.01	2.43	0.01	0.13	.03	0.01	2.43	0.01	0.12	.11	0.02	0.14	0.01	0.13	.09
Ta	0.01	0.30	0.01	0.16	.48	0.01	0.23	0.01	0.16	.14	0.01	0.30	0.01	0.03	.19
Zr	0.13	56.9	0.09	0.36	< .001	0.14	56.9	0.09	0.36	< .001	0.11	9.46	0.11	0.19	.04
**cerebrospinal fluid**															
Co	0.03	0.64	0.02	0.19	< .001	0.04	0.64	0.02	0.05	< .001	0.02	0.06	0.02	0.17	.33
Cr	0.28	2.05	0.23	2.62	.29	0.32	2.05	0.23	1.11	.04	0.22	0.97	0.29	2.62	.63
Mo	0.15	0.65	0.15	0.80	.86	0.15	0.54	0.16	0.69	.53	0.17	0.65	0.14	0.39	.33
Ni	0.69	2.82	0.59	7.83	.26	0.74	2.82	0.57	1.73	.13	0.61	2.04	0.61	7.83	.86
Ti	0.66	2.90	0.57	1.62	0.34	0.66	1.61	0.56	1.62	0.33	0.71	2.90	0.58	1.45	0.85
Al	9.95	55.0	10.5	38.0	0.90	9.94	36.6	10.3	38.0	0.98	10.8	55.0	10.6	16.0	0.47
V	0.16	0.89	0.12	0.58	0.12	0.16	0.65	0.13	0.58	0.43	0.19	0.89	0.13	0.40	0.06
Nb	0.01	0.26	0.01	0.17	0.78	0.01	0.26	0.01	0.10	0.29	0.01	0.05	0.01	0.17	0.19
Ta	0.01	0.01	0.01	0.01	-	0.01	0.01	0.01	0.01	-	0.01	0.01	0.01	0.01	-

© 2025 Rakow A et al. *JAMA Network Open*.

Zr	0.04	0.44	0.04	0.28	0.13	0.04	0.44	0.04	0.28	0.16	0.04	0.33	0.04	0.11	0.52

Abbreviations: CSF, cerebrospinal fluid; max, maximum metal level. [Wilcoxon matched-pairs signed rank test]

© 2025 Rakow A et al. *JAMA Network Open*.

eTable 8. *P* Values Resulting From the Spearman Correlation of Metal Levels in Whole Blood of Patients With at Least 1 Arthroplasty Implant in Situ

The p-value was corrected for multiple comparisons using the Bonferroni method. A p-value of 0.005(5) was considered statistically significant.

**Co**	0									
**Cr**	<0.001	0								
**Mo**	0.96	0.46	0							
**Ni**	0.05	0.004	0.78	0						
**Ti**	0.02	0.03	0.02	0.92	0					
**Al**	0.69	0.42	0.18	0.73	0.08	0				
**V**	0.001	0.11	0.76	0.67	0.03	0.18	0			
**Nb**	0.17	0.28	0.20	0.41	<0.001	0.69	0.03	0		
**Ta**	0.40	0.80	0.29	0.77	0.71	0.80	0.75	0.03	0	
**Zr**	0.002	0.003	0.70	0.47	0.21	0.53	0.10	0.78	0.81	0
	**Co**	**Cr**	**Mo**	**Ni**	**Ti**	**Al**	**V**	**Nb**	**Ta**	**Zr**

© 2025 Rakow A et al. *JAMA Network Open*.

eTable 9. *P* Values Resulting From the Spearman Correlation of Metal Levels in Serum of Patients With at Least 1 Arthroplasty Implant in Situ

The p-value was corrected for multiple comparisons using the Bonferroni method.

**Co**	0									
**Cr**	<0.001	0								
**Mo**	0.41	0.36	0							
**Ni**	0.54	0.09	0.07	0						
**Ti**	0.38	0.35	0.154	0.37	0					
**Al**	0.15	0.17	0.24	0.21	<0.001	0				
**V**	0.22	0.005	0.001	0.50	0.09	0.001	0			
**Nb**	0.51	0.58	0.44	0.11	0.13	0.15	0.51	0		
**Ta**	0.81	0.59	0.71	0.64	0.08	0.15	0.60	<0.001	0	
**Zr**	0.001	0.13	0.74	0.23	0.42	0.40	0.73	0.13	0.19	0
	**Co**	**Cr**	**Mo**	**Ni**	**Ti**	**Al**	**V**	**Nb**	**Ta**	**Zr**

© 2025 Rakow A et al. *JAMA Network Open*.

eTable 10. ***P*** **Values Resulting From the Spearman Correlation of Metal Levels in Cerebrospinal Fluid of Patients With at Least 1 Arthroplasty Implant in Situ**

The p-value was corrected for multiple comparisons using the Bonferroni method. A p-value of 0.005(5) was considered statistically significant.

**Co**	0									
**Cr**	<0.001	0								
**Mo**	0.14	0.97	0							
**Ni**	<0.001	<0.001	0.61	0						
**Ti**	0.07	0.59	0.150	0.01	0					
**Al**	0.14	0.008	0.47	0.88	0.33	0				
**V**	0.51	0.25	0.143	0.02	<0.001	0.31	0			
**Nb**	0.02	<0.001	0.39	0.02	0.77	<0.001	0.03	0		
**Ta**										
**Zr**	0.88	0.36	0.68	0.67	0.003	<0.001	<0.001	0.32		0
	**Co**	**Cr**	**Mo**	**Ni**	**Ti**	**Al**	**V**	**Nb**	**Ta**	**Zr**

© 2025 Rakow A et al. *JAMA Network Open*.

eTable 11. 90% Quantile (*$Q_{0.90}$*) of Metal Levels in Whole Blood and Serum of the Control Group and CSF Metal Levels With Ranges of Patients With Elevated Blood Metals (≥*$Q_{0.90}$*) and With at Least 1 Arthroplasty Implant in Situ

Metal levels are presented in µg/l.

		CSF levels control group				CSF levels implant group			
**analyte**	$Q_{0.90}$	min	median	max	IQR	min	median	max	IQR
**whole blood**									
Co	0.67	0.01	0.02	0.19	0.01	0.02	0.07	0.64	0.14
Cr	0.52	0.04	0.17	0.99	0.28	0.02	0.27	1.04	0.28
Ti	10.4	0.20	0.65	1.09	0.47	0.32	0.65	1.40	0.45
Nb	0.03	0.01	0.01	0.07	0.01	0.01	0.01	0.16	0.01
Zr	0.11	0.01	0.04	0.28	0.05	0.01	0.04	0.44	0.03
**serum**									
Co	0.27	0.01	0.02	0.19	0.02	0.02	0.04	0.64	0.07
Cr	0.54	0.04	0.24	0.77	0.29	0.02	0.26	1.05	0.32
Ti	5.65	0.13	0.57	1.10	0.34	0.12	0.75	1.40	0.40
Nb	0.05	0.01	0.01	0.03	0.01	0.01	0.02	0.16	0.05
Zr	0.16	0.01	0.04	0.28	0.02	0.01	0.05	0.44	0.02

Abbreviations: CSF, cerebrospinal fluid; IQR, interquartile range, $Q_{0.90}$, 90% quantile of the control

© 2025 Rakow A et al. *JAMA Network Open*.

eFigure 1. Cobalt and Chromium Levels of Patients With at Least 1 Cobalt-Chromium-Molybdenum Component and of Matched Control Participants

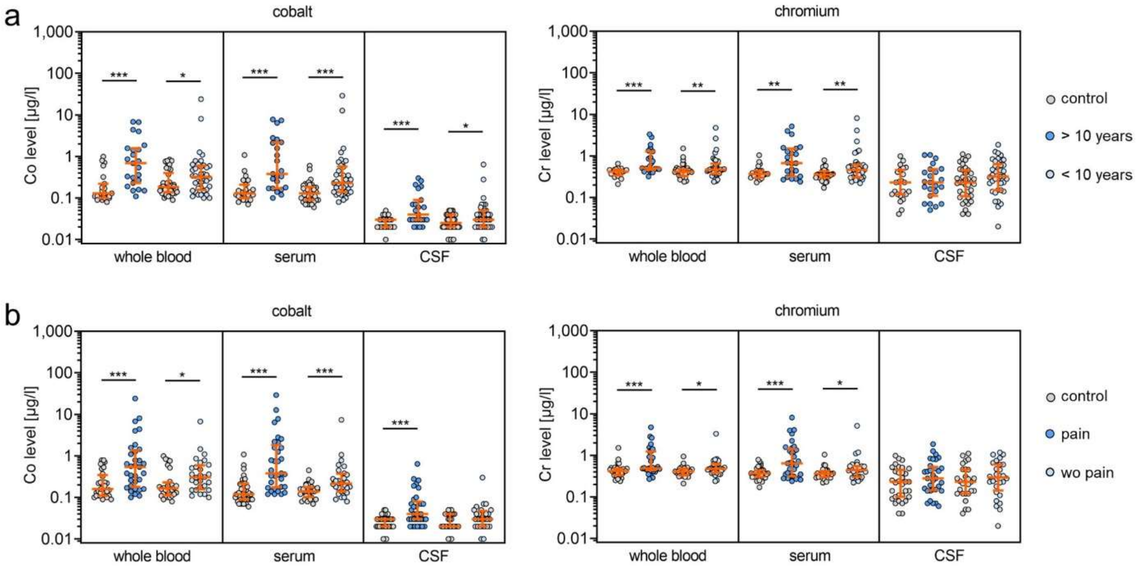

a) Comparison of Co levels and Cr levels in whole blood, serum and CSF of patients who had received their index implant more (n=23) and less (n=38) than 10 years before sampling with those of matched controls. (b) Comparison of Co levels and Cr levels in whole blood, serum and CSF of patients who had reported pain (n=33) in and no pain (n=28) the index joint with those of matched controls. [Wilcoxon matched-pairs signed rank test; median with IQR; levels of significance: *p < 0.05, **p < 0.01, ***p < 0.001].

© 2025 Rakow A et al. *JAMA Network Open*.

eFigure 2. Multimetal Quantification in Whole Blood, Serum, and CSF of Patients With Different Index Arthroplasty Implants

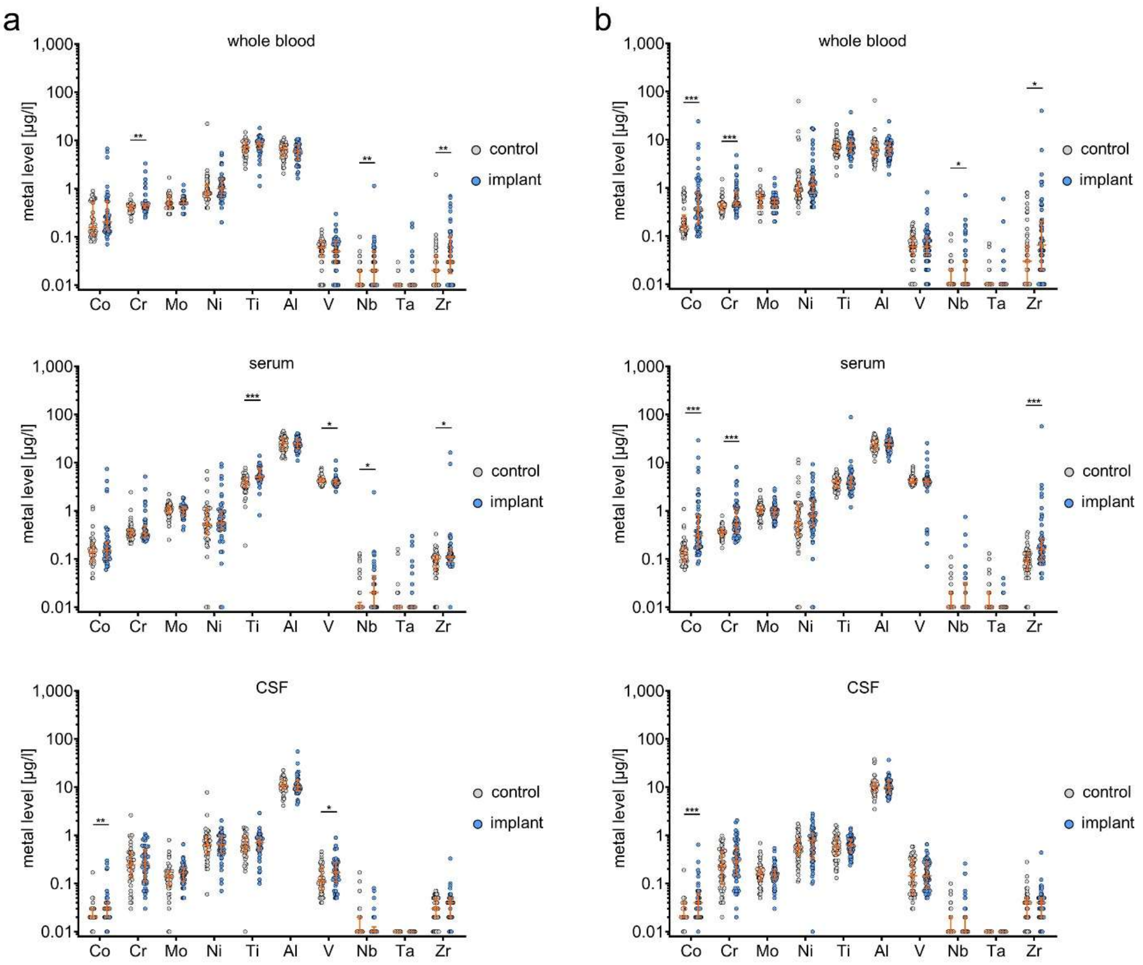

Levels of the quantified arthroplasty metals in whole blood, serum and CSF of patients with (a) hip arthroplasty implant (n=46) and (b) knee arthroplasty implant (n=54). [Wilcoxon matched-pairs signed rank test; median with IQR; levels of significance: *p < 0.05, **p < 0.01, ***p < 0.001].

© 2025 Rakow A et al. *JAMA Network Open*.

eFigure 3. Metal Levels of Patients in the Implant Group With Elevated Metals in Whole Blood and/or Serum and the Corresponding Levels in CSF

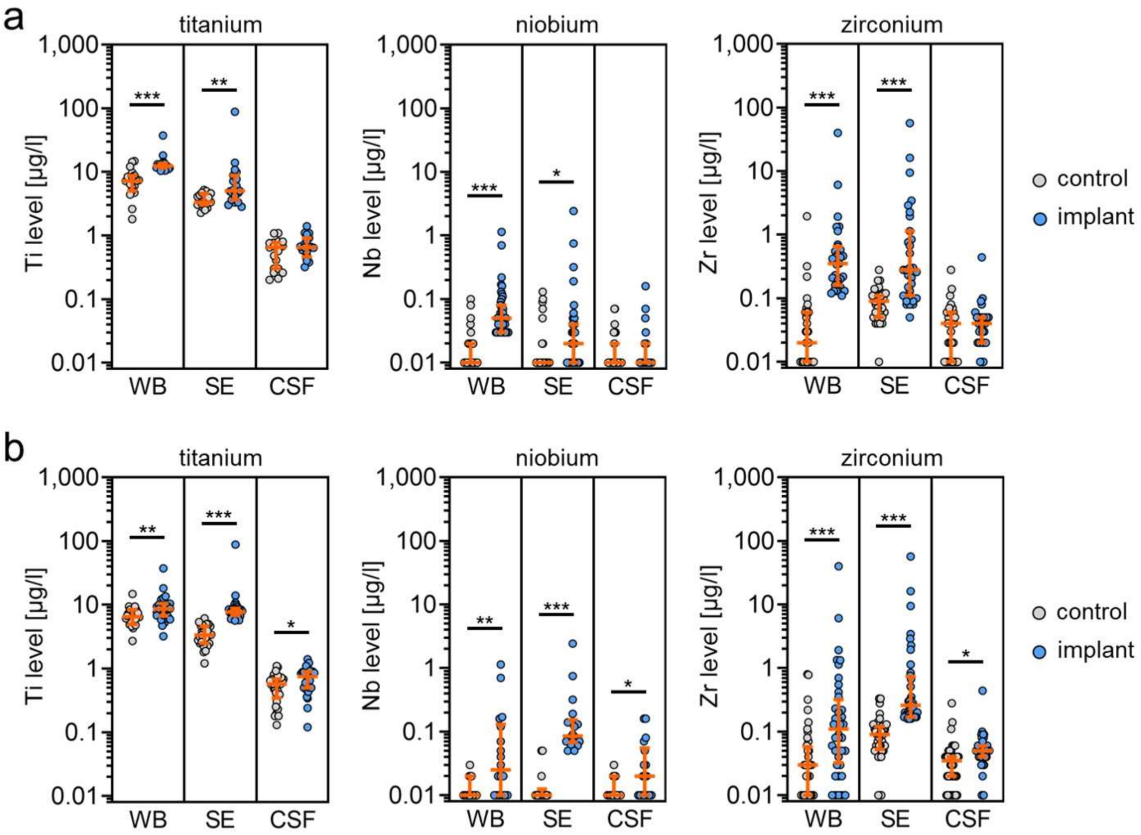

(a) Ti, Nb, and Zr levels of patients with at least one arthroplasty implant in situ whose metal levels in whole blood were above the 90% quantile of the control group (Ti, n=19; Nb, n=43, Zr, n=31) and metal levels of matched controls. (b) Metal levels of patients with at least one arthroplasty implant in situ whose serum metal levels are above the 90% quantile of the control group (Ti, n=29; Nb, n=18, Zr, n=40) and metal levels of matched controls. [Wilcoxon matched-pairs signed rank test; median with IQR; levels of significance: *p < 0.05, **p < 0.01, ***p < 0.001].

© 2025 Rakow A et al. *JAMA Network Open*.

eFigure 4. Intermatrix Correlation Analyses and Linear Regression of Log-Transformed Metal Levels of Patients in the Implant Group and Elevated Blood Metals

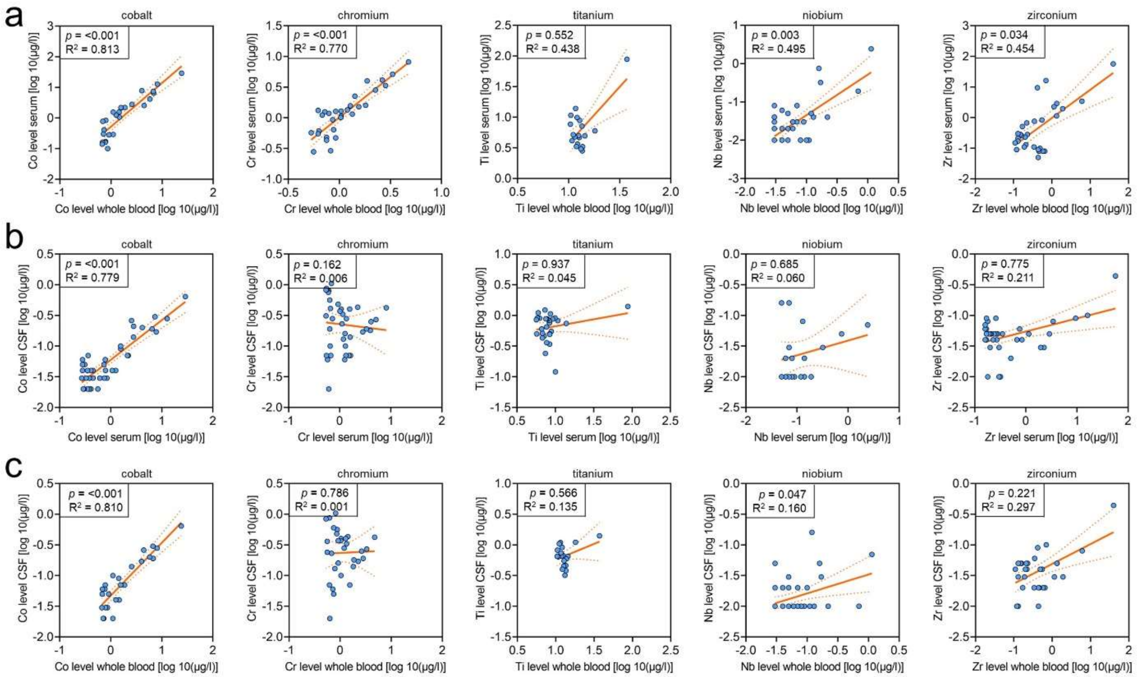

(a) Log-transformed metal levels of patients whose metal levels in whole blood are above the 90% quantile of the control group (Co, n=26; Cr, n=31; Ti, n=19; Nb, n=43, Zr, n=31) and the corresponding log-transformed metal levels in serum. (b) Log-transformed metal levels of patients whose metal levels in serum are above the 90% quantile of the control group (Co, n=41; Cr, n=38; Ti, n=29; Nb, n=18, Zr, n=40) and the corresponding log-transformed metal levels in CSF. (c) Log-transformed metal levels of patients whose metal levels in whole blood are above the 90% quantile of the control group (Co, n=26; Cr, n=31; Ti, n=19; Nb, n=43, Zr, n=31) and the corresponding log-transformed metal levels in CSF. [Linear regression

© 2025 Rakow A et al. *JAMA Network Open*.

eFigure 5. Zirconium Levels in Whole Blood, Serum, and CSF of 62 Patients With at Least 1 Cemented Arthroplasty Implant in Situ, 34 Patients With at Least 1 Arthroplasty Implant but No Bone Cement in Situ, and Matched Controls

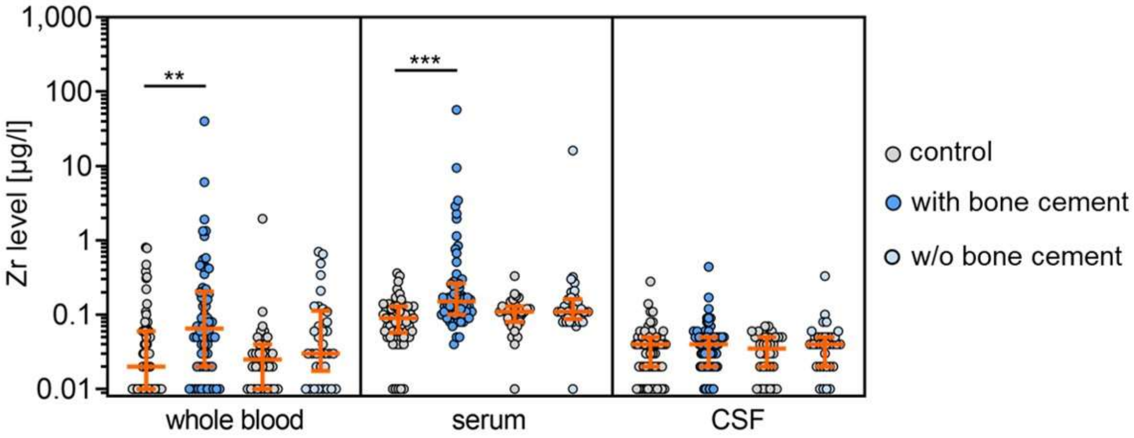

[Wilcoxon matched-pairs signed rank test; median with IQR; levels of significance: *p < 0.05, **p < 0.01, ***p < 0.001].

© 2025 Rakow A et al. *JAMA Network Open*.

eFigure 6. Serum S-100B Levels of (a) 102 Arthroplasty Implant–Naive Patients and 99 Patients With at Least 1 Arthroplasty Implant in Situ

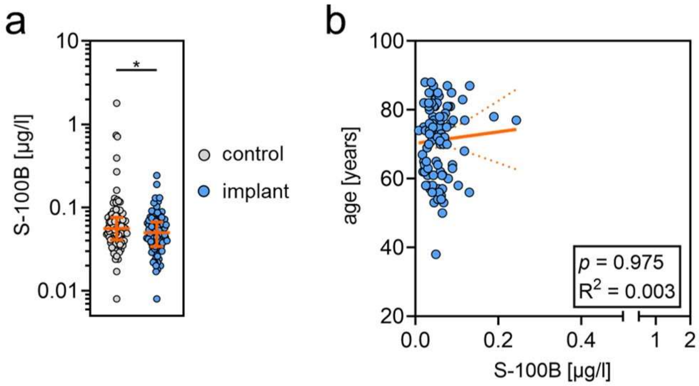

(n=99). [Wilcoxon matched-pairs signed rank test; median with IQR; levels of significance: *p < 0.05, **p < 0.01, ***p < 0.001]. Correlation of patient age and Serum S-100B levels with linear regression of the patients with at least one arthroplasty implant in situ (b) and of the arthroplasty implant naïve patients (c). [Linear regression with 95% confidence bands (dashed lines) and Spearman correlation analyses].

eFigure 7. Stratification of Patients According to Implant Status and Quantification of the Blood-CNS Barrier Integrity Marker S-100B

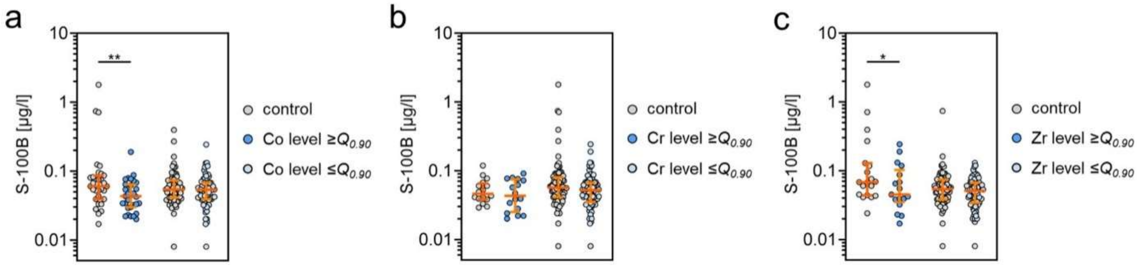

Serum S100-B of patients with at least one arthroplasty implant in situ whose (a) Co levels in CSF are above (n=31) and below (n=71) the 90% quantile of the control group and serum S-100B levels of matched controls, (b) Cr levels in CSF are above (n=16) and below (n=86) the 90% quantile of the control group and serum S-100B levels of matched controls, and (c) Zr levels in CSF are above (n=19) and below (n=83) the 90% quantile of the control group and serum S-100B levels of matched controls. [Wilcoxon matched-pairs signed rank test; median with IQR; levels of significance: *$p < 0.05$, **$p < 0.01$, ***$p < 0.001$].

© 2025 Rakow A et al. *JAMA Network Open*.
