# Supplementary material for: Metal Concentrations in Blood and Cerebrospinal Fluid of Patients With Arthroplasty Implants
Source: JAMA Netw Open. 2025 Mar 28;8(3):e252281. doi: 10.1001/jamanetworkopen.2025.2281 (PMC11953760; doi:10.1001/jamanetworkopen.2025.2281)
Supplement: Supplement 2. — Data Sharing Statement [file jamanetwopen-e252281-s002.pdf]

Data Sharing Statement

Rakow. Metal Concentrations in Blood and Cerebrospinal Fluid of Patients With Arthroplasty Implants. *JAMA Netw Open*. Published March 28, 2025.
doi:10.1001/jamanetworkopen.2025.2281

Data
Data available: Yes
Data types: Deidentified participant data
How to access data: Data will be made available after publication of the manuscripts via 1) http://drks.de/search/en/trial/DRKS00014555; 2) http://drks.de/search/en/trial/DRKS00014556; and 3) if reasonable request is sent to anastasia.rakow@med.uni-greifswald.de, janosch.schoon@med.uni-greifswald.de or carsten.perka@charite.de
When available: With publication

Supporting Documents
Document types: Statistical/analytic code, Informed consent form, Other (please specify)
Additional Information: CRF
How to access documents: http://drks.de/search/en/trial/DRKS00014555 ; http://drks.de/search/en/trial/DRKS00014556
When available: With publication

Additional Information
Who can access the data: anyone requesting the data
Types of analyses: for a specified purpose
Mechanisms of data availability: after approval of a proposal, or with a signed data access agreement
